# Users evaluating physical, virtual, and mixed reality prototypes exhibit differential DLPFC brain activity

**DOI:** 10.1038/s41598-025-23557-z

**Published:** 2025-11-12

**Authors:** Henrikke Dybvik, Christopher Cox, Isabelle Ormerod, Pasi Aalto, Chris Snider

**Affiliations:** 1 University of Bristol, Bristol, UK; 2 Norwegian University of Science and Technology, Trondheim, Norway

**Keywords:** Prototyping, Mixed reality, Head-mounted display, Virtual reality, User study, fNIRS, Decision, Mechanical engineering

## Abstract

The emergent use of mixed reality (MR) technology in product development and evaluation provides new opportunities for prototyping across the physical and virtual domain. However, it is unknown how MR prototypes affect users’ cognitive processes during final stage product evaluation. This experiment explores how different prototype representations—physical, virtual, and MR—affect users’ DLPFC brain activity as measured by functional near-infrared spectroscopy (fNIRS), subjective workload, and affective state. N_total_=88 participants split in three independent groups received either a physical (*N* = 30), virtual (*N* = 30), or MR (*N* = 28) representation of a power drill, tasked to evaluate its usability and propose a design change. The results demonstrate significantly different oxygenated and deoxygenated haemoglobin concentration changes in users’ dorsolateral prefrontal cortex (DLPFC), depending on representation mode (i.e., physically, virtual, or MR), and task (i.e., evaluation or proposing a design change). While there were some between-group differences in physical demand and subjective performance, other subjective workload and affective state measures did not significantly differ. These results are novel, demonstrating that users’ DLPFC brain activity differ depending on prototype representation mode. This could imply differing cognitive processes, but further research is required. Nevertheless, choosing prototype representation mode is not trivial and should be a careful consideration.

**Supplementary Information:**

The online version contains supplementary material available at 10.1038/s41598-025-23557-z.

## Introduction

Product prototyping requires the confluence of many media, tools and technologies to create an output^1^. A prototype can be defined as any representation of a product prior to its final form^2^, with purposes including ideation, design refinement, evaluation, and communication^3^. In this sense, prototyping is a learning activity^3–6^, informing and evolving design thinking processes as ideas are refined. Typically, designers select prototyping material (media) via intuition^4,7,8^, despite the inherent affordances that each media holds^9^. At a fundamental level, prototyping media can be typed on a range from fully physical to fully digital media (termed their *domain*)^10–12^, with each domain holding its own limitations and advantages. For example, physical prototypes are tactile and can prove a “feels-like” experience^1,2,10^, but can be expensive and time-consuming to change^1,10,13^, whereas virtual prototypes can easily be changed (provides inexpensive flexibility)^1,10^, but are intangible. While some researchers formally consider the relative benefit of each domain and its influence on prototyping outcomes, this is relatively under-studied^9,10^.

Recent technological developments have also introduced the possibility of Mixed Reality (MR) prototyping, whereby physical and digital are seamlessly blended via immersive visuals (i.e. virtual reality) and spatial interaction^10,14–16^. MR allows combining the advantages of the physical and virtual and mitigating their limitations^10,16,17^. For example, an MR prototype could provide users with a full tactile experience of the product in combination with virtual flexibility displaying an interactive interface. While studies have shown that MR prototypes, to some degree, influence users’ subjective perceptions and evaluations of the product, as well as objective performance metrics^18–20^, current applications of MR prototypes remain ad hoc^10^, with little establishment of best practice compared to traditional media.

As prototyping is a thinking skill, and the use of physical and virtual media affect cognitive decision-making processes, we hypothesise that MR prototypes similarly affect the cognitive processes governing decision-making. This hypothesis is further supported by foundational neuroscience research showing that the human brain responds differently to stimuli presented in two and three dimensions^21,22^. There is minimal research investigating links between prototyping media and cognitive processes from a neurological perspective, and no current research investigating (potential) changes in cognitive processes in users evaluating prototypes as a result of the domain used for the prototype (i.e., physical, virtual, and MR).

In this context, and where MR technology is becoming increasingly accessible and capable, it is valuable to understand if the impact of prototyping media choice extends beyond inherent affordances related to artefact (i.e. fidelity, flexibility) or process (i.e. time and monetary cost) and into cognitive behaviours of the designers themselves. Should such be observed even when prototypes are as aligned as viable across domains, and should variation in cognitive behaviour exist across prototyping activities, then further research into the neurocognitive influence of media should be conducted.

This work explores how different prototyping domains (physical, virtual, and MR) affects users brain activity when evaluating final stage prototypes. Our research questions are:

1. How does different prototyping media affect the brain activity of users evaluating prototypes?
  Hypothesis (non-directional): There is a difference in brain activity between different domains (MR, physical, virtual).
2. How does different prototyping media affect users workload, affective state, and stress when evaluating prototypes?
  Hypothesis (non-directional): There is a difference in workload, affective state, and stress between different domains (MR, physical, virtual).

We designed an experimental study wherein *N* = 88 participants were tasked with a product evaluation task of a final stage prototype of a power drill. Participants were split into three independent groups, receiving a prototype represented Physically (*N* = 30), Virtually (*N* = 30) or with MR (*N* = 28). Using the prototype, their task was to (a) evaluate the drills usability considering its task (users would use the drill as a private person, using it to assemble IKEA furniture and hang up pictures), and (b) to propose a design change. To obtain brain activity measures we collected functional near-infrared spectroscopy (fNIRS) data, which provides measurements of concentration changes of oxygenated (HbO) and deoxygenated (HbR) haemoglobin, of participants dorsolateral prefrontal cortex (DLPFC), throughout the experimental procedure. Subjective measures of workload (NASA Task Load Index^23^ and Overall Workload^24^, affective state^25^, and stress level were collected post experiment. The results evidence brain activity differences between prototypes represented physically, virtually, and with MR. We also found evidence for differential brain activity associated with different task (i.e., evaluation, and proposing a design change). This could imply that prototype representation mode affects users’ cognitive processes, however, more research is required for corroboration.

## Results

### Ensuring balanced groups

A chi-squared test showed there were no significant differences between groups in terms of biological sex (χ²(2, *N* = 88) = 0.08, *p* = 0.9621) and the number of participants that were students (χ²(2, *N* = 88) = 5.58, *p* = 0.06153). Fishers exact test showed there were no significant difference in handedness (*p* = 0.6003) between groups. A bootstrap version of the heteroscedastic one-way ANOVA for trimmed means (Table 1) showed there were no significant differences between groups in design experience, VR experience, CAD experience, experience with physical prototyping, and experience with using a real drill. For age, there was a significant difference between Physical and MR, but not for other contrasts.

This suggests groups were balanced on all individual variables (biological sex, ratio of student participants, handedness, and experience with design, VR, CAD, physical prototyping, and using a real drill), apart from age.

**Table 1:** Between-group analysis on individual demographical variables. This table provides the pairwise comparisons.

Demographic variable	Contrast	Virtual vs. physical	Virtual vs. MR	Physical vs. MR
Sex	p-value^1^	1	1	1
Student (yes/no)	p-value^1^	0.8847	1	0.1087
Handedness	p-value^2^	1	1	0.660
Age	$\psi$	3.83	− 2.67	− 6.50
	95% CI	[− 1.83, 10.61]	[− 9.44, 4.83]	[− 12.39, − 0.89]
	Effect size^3^	0.311	0.311	0.311
Design experience (years)	$\psi$	0.72	0.56	− 0.17
	95% CI	[− 2.17, 5.50]	[− 3.11, 5.33]	[− 3.56, 2.72]
	Effect size^3^	0.089	0.089	0.089
VR experience	$\psi$	− 0.39	− 0.94	− 0.56
	95% CI	[− 2.28, 1.17]	[− 2.83, 0.72]	[− 2.67, 1.67]
	Effect size^3^	0.167	0.167	0.167
CAD experience	$\psi$	− 0.44	1.28	1.72
	95% CI	[− 3.22, 2.67]	[− 1.28, 3.94]	[− 1.17, 4.22]
	Effect size^3^	0.185	0.185	0.185
Physical prototyping experience	$\psi$	− 0.94	0.67	1.61
	95% CI	[− 4.06, 2.17]	[− 1.89, 3.22]	[− 1.22, 4.44]
	Effect size^3^	0.174	0.174	0.174
Real drill experience	$\psi$	0.11	0.78	0.67
	95% CI	[− 2.06, 2.39]	[− 1.78, 3.28]	[− 1.94, 2.83]
	Effect size^3^	0.101	0.101	0.101

^1^Pairwise Chi-squared tests with a Bonferroni correction.

^2^Pairwise Fisher’s exact test with a Bonferroni correction.

^3^Effect size for omnibus robust test (WRS2::t1waybt, i.e., not for individual contrasts).

### fNIRS results

#### Main effect of age

A robust mixed-effects model found a significant main effect of age on activation in one HbO channel (S2-D1, *beta =* 0.281, *SE =* 0.0797, *t =* 3.525, *dfe =* 174, *q =* 0.01948). See Supplementary Materials for statistics for all channels.

#### GLM1: between-group differences in overall activation (i.e., including both tasks)

**Fig. 1:**
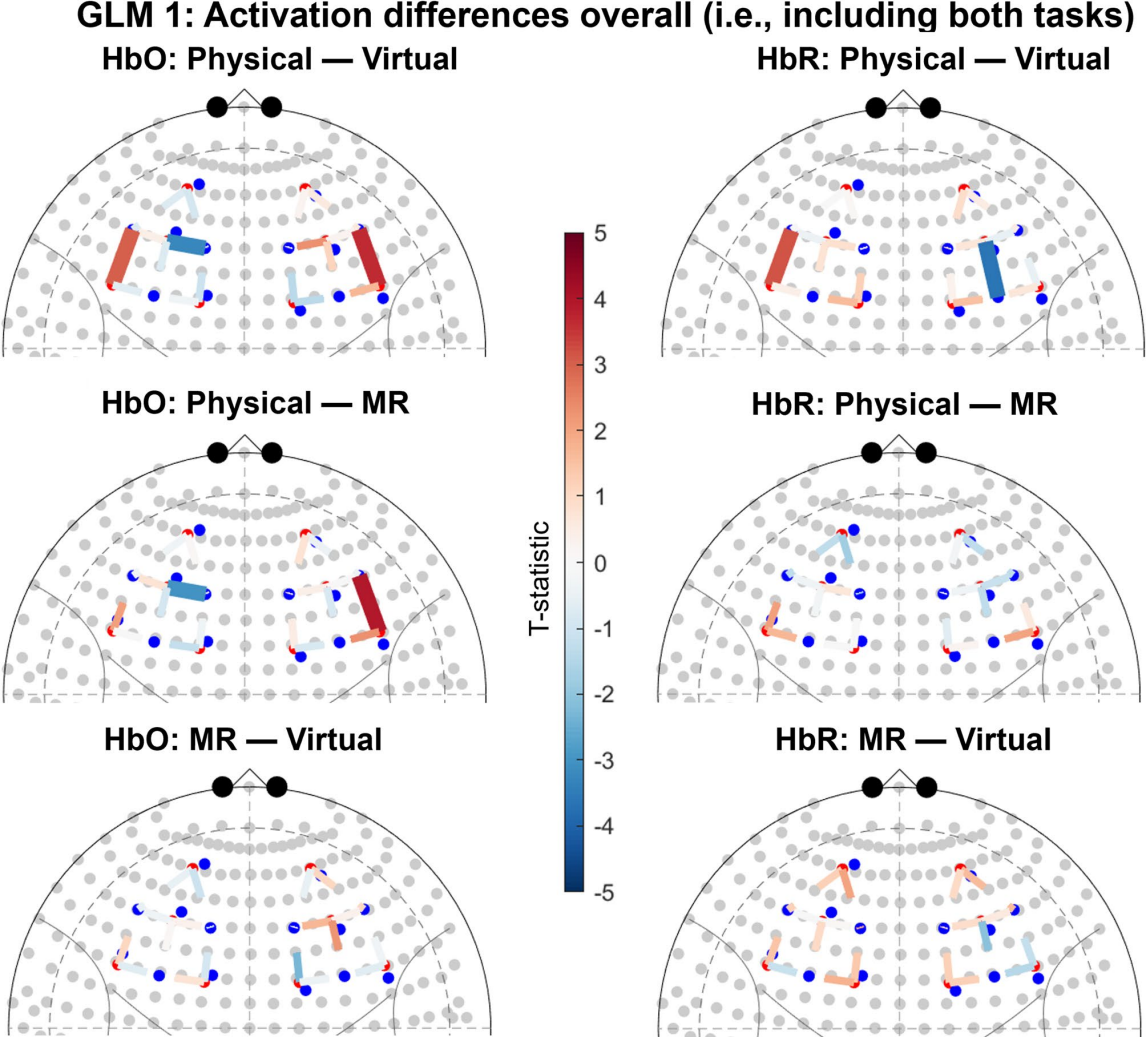
Results of group contrasts for overall activation (i.e., including both tasks). Significant channels (q < 0.05) are shown as sold lines. Channels are displayed on top of the 10–20 coordinate system. For HbO contrasts, positive t-values (red) correspond to relatively larger activation for the first term of the contrast, and negative t-values (blue) correspond to larger activation for the second term. The opposite pattern applies to HbR contrasts. The t-statistic is scaled to [− 5, 5].

Between-group contrasts based on group level activation resulting from a mixed-effect model including both tasks yielded several significant channels, see Fig. 1. For Physical compared to Virtual, there was a significant HbO increase in two channels, HbO decrease in one channel, as well as a HbR increase in one channels, and HbR decrease in one channel. This indicates higher activation in certain regions and lower activation in other regions in Physical compared to Virtual. For Physical compared to MR there was a significant HbO increase in one channel and HbO decrease in one channel. This indicates higher activation in certain regions and lower activation in other regions in Physical compared to MR. There were no significant differences between MR and Virtual. See Supplementary Materials for detailed statistics.

#### GLM2: between-group differences in activation during evaluation task

**Fig. 2:**
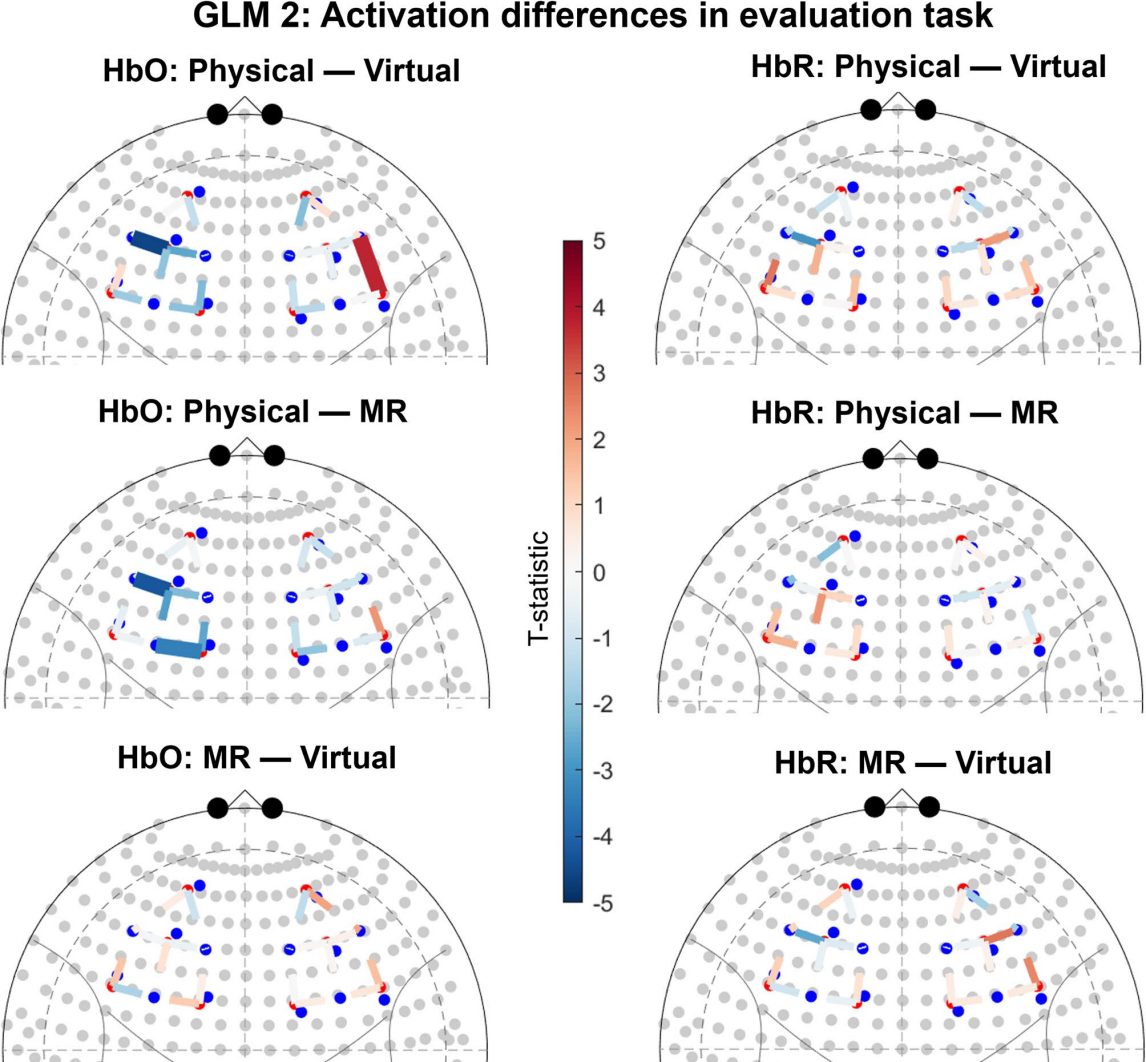
Results of group contrasts for evaluation task. Significant channels (q < 0.05) are shown as sold lines. Channels are displayed on top of the 10–20 coordinate system. For HbO contrasts, positive t-values (red) correspond to relatively larger activation for the first term of the contrast, and negative t-values (blue) correspond to larger activation for the second term. The opposite pattern applies to HbR contrasts. The t-statistic is scaled to [− 5, 5].

Between-group contrasts based on group level activation resulting from a mixed-effect model including only the evaluation task yielded several significant channels, see Fig. 2. For the contrast comparing Physical to Virtual we found significant HbO increase in one channel (right hemisphere) and significant HbO decrease in one channels (left hemisphere). This indicates higher activation in the left hemisphere and lower activation in the right hemisphere in Virtual compared to Physical. For the contrast comparing Physical to MR we found significant HbO decrease in two channels (left hemispheres) and significant HbR increase in one channel (left hemisphere). This indicates higher activation in MR compared to Physical. There were no significant differences between MR and Virtual. See Supplementary Materials for detailed statistics.

#### GLM3: between-group differences in activation during design change task

**Fig. 3:**
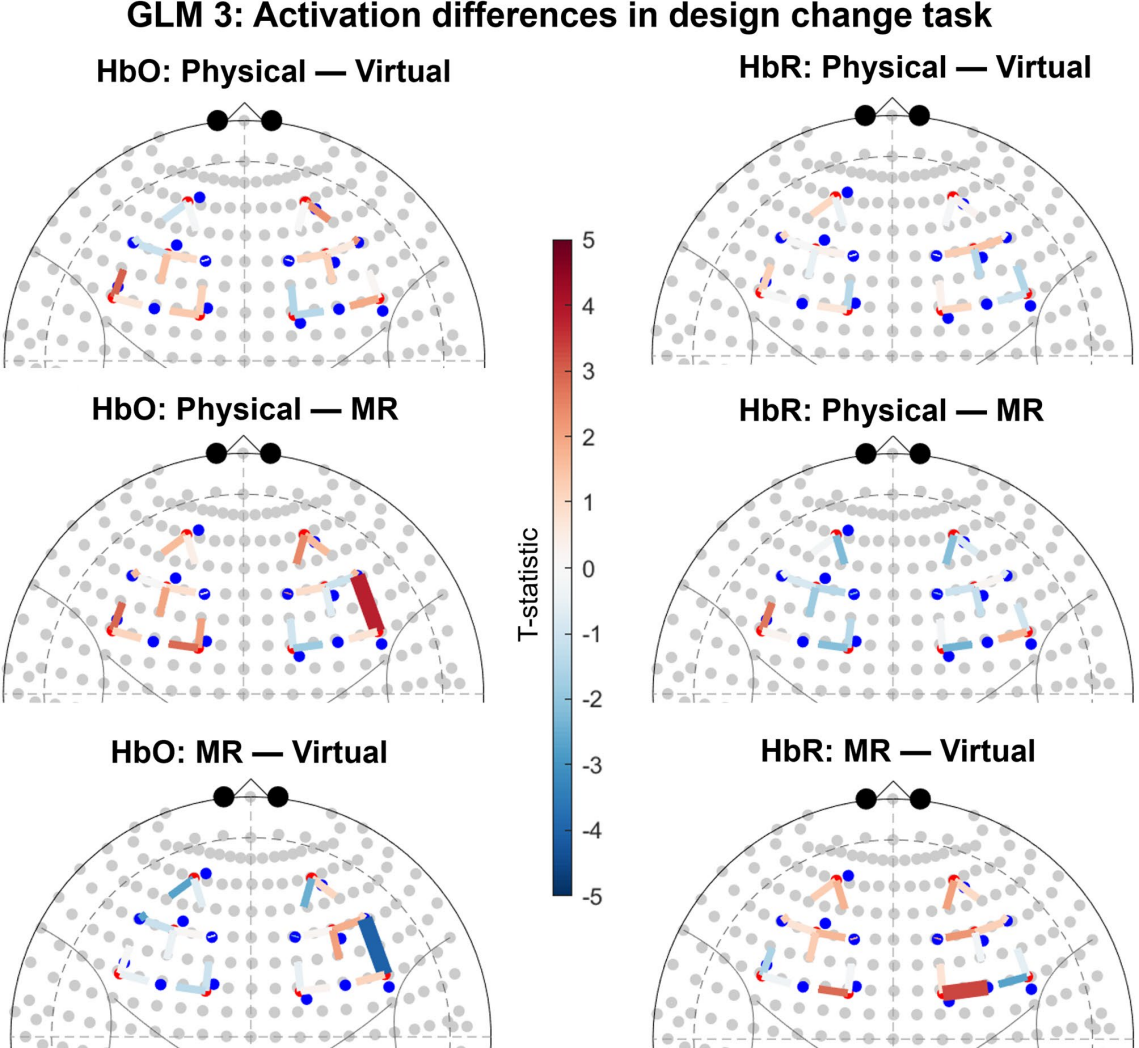
Results of group contrasts for design change task. Significant channels (q < 0.05) are shown as sold lines. Channels are displayed on top of the 10–20 coordinate system. For HbO contrasts, positive t-values (red) correspond to relatively larger activation for the first term of the contrast, and negative t-values (blue) correspond to larger activation for the second term. The opposite pattern applies to HbR contrasts. The t-statistic is scaled to [− 5, 5].

Between-group contrasts based on group level activation resulting from a mixed-effect model including only the design change task yielded several significant channels, see Fig. 3. For the contrast comparing Physical and MR we found a significant HbO increase in one channel (right hemisphere). This is indicative of higher activation in Physical compared to MR. For the contrast comparing MR to Virtual we found a significant HbO decrease in one channel and a significant HbR increase in one channel. This indicates higher activation in Virtual compared to MR. There were no significant differences between Physical and Virtual. See Supplementary Materials for detailed statistics.

#### Workload and affective state results

**Fig. 4:**
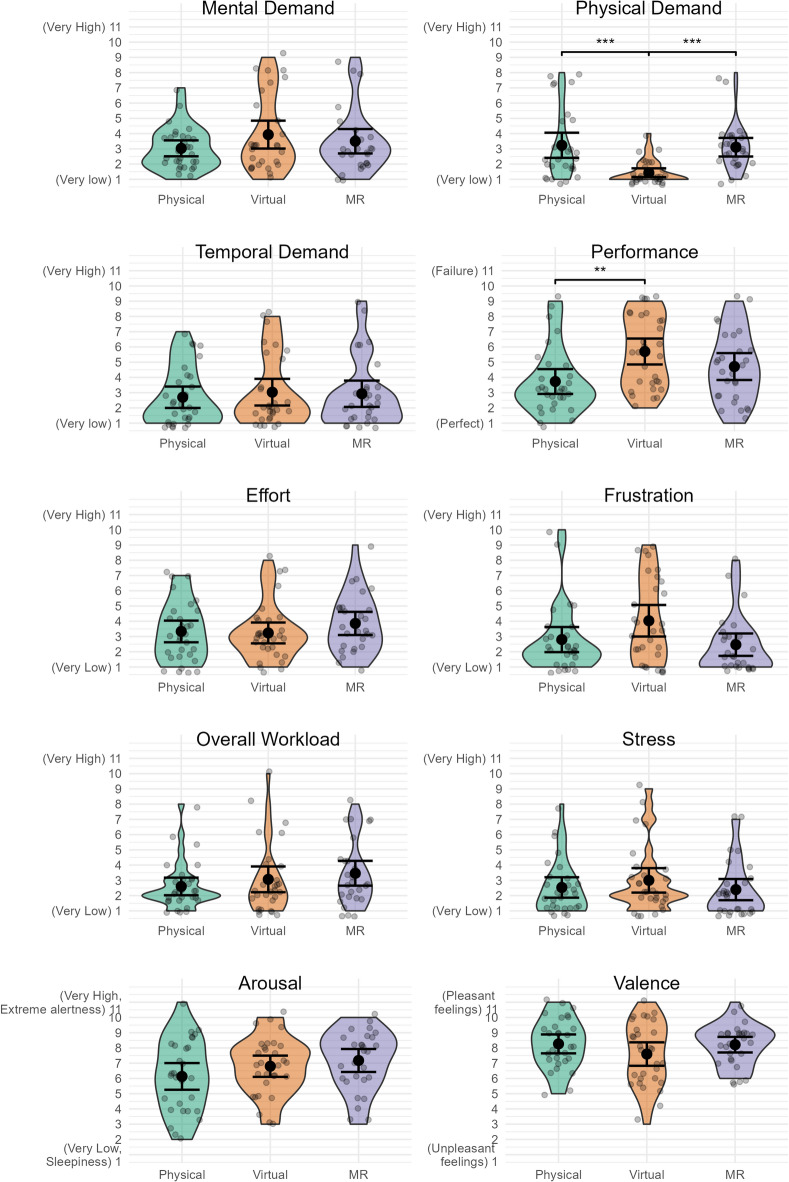
Violin and scatterplots of participants subjective ratings of workload (NASA Task Load Index dimensions and Overall Workload), affective state and stress. The error bar represents 95%CI assuming normality, centred around the mean. *p ≤ 0.05, **p ≤ 0.01, ***p ≤ 0.001.

A linear model or, in case of violated assumptions, a bootstrap version of the heteroscedastic one-way ANOVA for trimmed means, showed no significant differences in mental demand, temporal demand, effort, frustration, overall workload, stress, arousal, and valence between groups. There was a significant difference in performance between Physical and Virtual (*∆M* = -1. 97, %CI[-3.38, -0.55], *t*(85) = -3.39, *p =* 0.003), but no significant differences for other contrasts. See Supplementary Materials for statistical details. For physical demand, the linear model whose assumptions was not met (non-normality in residuals) and the robust model, yielded different results (see Supplementary Materials for details). We therefore opted to use the Kruskal–Wallis rank sum test and post-hoc pairwise comparisons with the Dunn test. The Dunn test found significant differences between Physical and Virtual (*Z =* 4.13, *p <* 0.001), and Virtual and MR (*Z =* 4.74, *p <* 0.001), but no significant difference between Physical and MR. This corroborates what we see visually in Fig. 4.

## Discussion

There was a significant difference in age between groups, reflecting that groups were not perfectly matched on age. However, the effect size and beta value was small, indicating that this effect was minor. We therefore conducted an analysis checking whether there was a main effect of age on haemoglobin concentrations (i.e., brain activation as measured by fNIRS), which yielded one significant channel. Therefore, the main analysis controlled for age as a random variable (in addition to subject), which, together with the small effect size, should limit age’s effect on the results.

Overall, including both the evaluation and the design change task, there were significant differences in brain activation between Physical and Virtual, and Physical and MR. Specifically, higher activation in bilateral BA44 and right DLPFC, and lower in parts of left DLPFC in Physical compared to Virtual. In left BA44 the was a significant increase in both HbO and HbR. Physical yielded lower activation in left dlPRC and higher in right BA44 compared to MR. There were no overall differences between MR and Virtual. When considering only the evaluation task, there was higher activation in right BA44 and lower activation in left DLPFC when comparing Physical and Virtual. There was significantly lower activation in the left DLPFC in Physical compared to MR (i.e., MR yielded higher activation than Physical). Again, there were no significant differences between MR and Virtual. When considering only the design change task, a slightly different pattern emerged. Here, there were no significant differences between Physical and Virtual. Physical yielded higher activation than MR in right BA44. MR yielded lower activation than Virtual in right BA44, and right DLPFC (i.e., Virtual yielded higher than MR in the right hemisphere).

For subjectively reported workload, affective state and stress there were no differences between groups, apart from (subjective) performance and physical demand. Participants felt they performed significantly better at conducting the experimental task in Physical compared to Virtual, although the mean difference of approximately 2 on an 11-point scale was relatively small. Physical demand was significantly lower in Virtual compared to the other conditions. The difference in physical demand was expected as the conditions were different in terms of physical demand. This serves as a confirmation that product evaluation using a digital model on a computer is less physically demanding than physically handling an object. It should further be noted that ratings of physical demand were relatively low for all groups.

In the following discussion we focus on our region-of-interest, the DLPFC, which the study was set up to investigate. Significant between-group differences in BA44 will not be discussed. For an evaluative context there was no difference in brain activity between MR and Virtual, suggesting little differences in participants brain activity when evaluating prototypes represented by MR and virtually. Participants evaluating physical prototypes exhibited lower brain activity in the left DLPFC than participants evaluating MR prototypes. This could indicate that physical prototypes placed smaller demands on participants working memory (WM), resulting in lower cognitive effort, or alternatively that cognitive effort was lower regardless of WM demands. Because the left DLPFC produces stimuli responses drawing directly upon environmental cues^26^, this result could suggest that physical prototypes gave more environmental cues, whereas participants evaluating MR prototypes could rely less upon environmental cues, thus yielding higher brain activation in that region. An increased ability to rely on environmental cues could also result in lower cognitive effort. Though there were no significant differences in subjective workload, MR participants reported slightly higher overall workload, as well as higher ratings on most individual NASA-TLX workload dimensions (mental demand, temporal demand, subjective performance, effort), supporting the brain activity results. Of course, the suggested cognitive processes underlying these differences are speculations and further research is needed to corroborate or reject these explanations. There was a similar difference, i.e., lower brain activity in the left DLPFC, for participants evaluating physical prototypes compared to virtual prototypes. The suggested explanation above could apply for this group comparison as well, as there are fewer environmental cues in a virtual than a physical prototype.

For a design change context, there were no brain activity differences between participants considering physical and virtual prototypes, suggesting little differences in participants proposing design changes to physical and virtual prototypes. Participants proposing design changes to MR prototypes exhibited lower activation in right DLPFC than participants proposing design changes to virtual prototypes; i.e., in a context where participants are proposing design changes, virtual prototypes yielded higher brain activity in right DLPFC than MR prototypes. Proposing design changes is an open-ended process, in this context without objectively correct answers, and it involves exploring different alternatives, considering design options, and deciding on one amongst multiple options. Such inductive thinking is lateralized to the right DLPFC^26^. It could be that attempting to come up with a design change to virtual prototypes elicits a greater need for inductive thinking than MR prototypes, due to the lack of tactility and therefore that there are less environmental cues to draw upon. Again, further research is needed to corroborate or reject this explanation.

As far as the authors are aware, there are no other fNIRS product evaluation studies^27^ or any other neuroimaging studies that are investigating the effect of the domain used to represent a prototype. Therefore, there is no other research to compare our results to. We hope this study can serve as a foundation for future studies to come on this topic.

Our first hypothesis—there is a difference in brain activity between different domains—is accepted as it is supported by the results presented here. The results suggest there are differences in people’s brain activity when they evaluate final stage prototypes depending on prototype representation mode. Wherein these differences lie and the magnitude thereof depends on which representation modes are compared. Whether or not certain regional brain activity in a certain representation mode is better than another is highly dependent on context, and remains to be determined. We have demonstrated existence of such differences. Under the assumption that these brain activity differences are valuable or/and meaningful, we now need to investigate which regional brain activity is better (or worse) in which context using which representation mode. This investigation must of course account for the aim and outcome of the product evaluation process, i.e., investigate what, if any, practical effect these brain activity differences have on participant decision-making. The key takeaway is that there are differences in brain activity and thus possibly cognitive processes depending on the choice of representation mode for prototype evaluation with users. The choice of representation mode is thus not trivial and should be a careful consideration.

Our second hypothesis—there is a difference in workload, affective state, and stress between different domains—is largely rejected as we found no differences in workload, affective state or stress levels between prototype representation modes, with the exception of physical demand and subjective performance (which had minor differences). With the exception of physical demand and subjective performance we accept the alternate hypothesis: there is no difference in subjectively evaluated workload, affective state and stress between participants evaluating physical, virtual and MR prototypes.

This study comes with limitations. Although participants were randomly allocated to groups, the age of groups could have been better balanced to eliminate age’s potential influence. This was not possible because participants were assigned to groups upon arrival and demographics were collected after experiment completion. It seemed like some participants in the Virtual group did not realise they could interact with the digital model, because they did not do anything with it apart from visual inspection. Alternatively, the participants did not interact with the virtual model because they did not see the need to, think, or want to engage in any interaction. The MR condition likely brings an aspect of novelty to it, which might have increased task duration for both evaluation and design change, and possibly workload ratings. Other researchers found a “fascination effect” in MR ^18^. Although we did not record a similar measure, this effect could be present in our experiment. This novelty might have influenced the fNIRS data. The fNIRS data included reading and understanding the task. In future research, changes to the instructions could separate the time reading and understanding the task from the time conducting the task, providing a more equal data basis for comparing conditions/groups. We must investigate whether participants subjective evaluation of the prototypes differ. This is not within the scope of this article, but will be subject to further work. Decision-making is a highly complex cognitive process relying not only on the DLPFC but also on other frontal lobe regions and deeper structures of the brain, which our 8 × 8 optode setup cannot capture.

The experiment utilises the explorative “lab in the wild” approach called for by Vigliocco et al.^28^ and Matusz et al.^29^ by specifically targeting design processes in three affordances with complex stimuli as opposed to individual factors within those processes. The limitation of this approach is that individual factors (visual perception, haptics, attention, etc.) affecting brain activation cannot be separated, but must all be understood as an integral part of the task.

The extent to which the results can be generalised to apply to other prototypes can be discussed. Our prototype was a DeWalt power drill, a product that the vast majority of our participants were familiar with. It could be that the results are not generalisable to products far from the hand tool category and that the results would have been different if participants were unfamiliar with the product. The technical complexity of the product, and the prototype stage (i.e., later stage vs. early-stage prototypes) could possibly also have an influence on the results, and should be investigated in future research. While participants might have been affected by the DeWalt branding, this was consistent across groups.

In summary, there are differences in DLPFC brain activity between users evaluating and proposing design changes to final stage prototypes represented physically, digitally, and with MR, as evidenced by HbO increase and HbR decrease as measured by fNIRS. In other words, users DLPFC brain activity differ depending on the prototype’s representation domain. This could imply differing cognitive processes. The practical implications of this remains to be determined. Future work should investigate and, if possible, establish functional links between the evidenced brain activity and behaviour or output, i.e., investigating whether participants are using different cognitive strategies, abstraction levels, or evaluation heuristics depending on prototype representation domain. This would support superior media selection and improved prototyping outcomes. However, we encourage practitioners to recognize and consider these brain activity differences when selecting representation mode for a prototype, whether that be MR, virtual, or physical.

## Methods

**Fig. 5:**
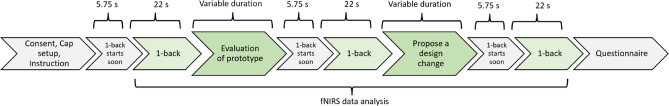
Experimental procedure. Virtual Reality headset uses Quest Link virtual desktop to read and interact with instructions.

We conducted an experiment comparing physical, virtual, and mixed reality prototyping technologies that included evaluative and proactive steps. It was a between-group experimental design with three groups: physical, virtual, and mixed reality (MR), that all performed the same tasks. Figure 5 depicts the experimental procedure. Participants had two primary tasks that were self-paced, (a) performing a final stage prototype evaluation of a power drill, and (b) propose a change to the design of the prototype, i.e., drill. These tasks embody two primary purposes of design prototyping (active learning and design refinement) thereby aligning the tasks to realistic prototyping activity^7^. These tasks are described in detail below. A word 1-back task was included as a secondary task to provide a baseline for brain activity measurements. The 1-back block (Fig. 5) included a 5 s presentation screen (“1-Back memory task will soon begin. Press < SPACE > if the letter is similar to the previous one.“), a 0.75 s pause, followed by a task duration on of 22 seconds. PsychoPy v2023.2.3 ^30^ was used to present instructions, the 1-back, the evaluation task and the design change task, and gather responses to a questionnaire. Participants used a standard keyboard and mouse to navigate the self-paced instructions.

### Experimental procedure

Participants were informed this was a product evaluation study where they would interact with a prototype of a product that was presented through a media ranging from fully physical to fully virtual. Individually sized caps were fitted with fNIRS sensors upon participants arrival. After providing written informed consent, participants were outfitted with the fNIRS cap. Afterwards, written instructions presented through PsychoPy guided participants through the experiment. Participants first performed the prototype evaluation task, followed by the design change task. 1-back tasks were completed before, between, and after these tasks.

### Task and conditions

Prototype evaluation: Participants were tasked to perform a final stage prototype evaluation of a power drill. Participants were informed they would be using the drill as a private person, using the drill to hang up pictures on a wall, and assemble IKEA furniture. They could interact with the prototype while performing the evaluation. Typically this activity is considered active learning, where direct prototype interaction informs the user’s views of its function, form, and behaviour^7^. Design change: After prototype evaluation participants were asked to propose one change they would make to the design of the drill. Participants were asked to articulate (explain by speaking out loud) the design change. They were still allowed to interact with the prototype. This activity typically requires proactive ideation or design exploration, with the user forming refinements of the prototype towards a subjective ideal functional, form, or behavioural state^7^. See Supplementary Materials for complete task instructions. Before and between tasks, participants performed a 1-back memory task, which was implemented to baseline brain activity recordings.

**Between-group experimental design**: Participants were allocated to one of three independent groups: Physical, Virtual, and Mixed Reality (MR). In the Physical group, participants received a real power drill, a cordless DeWalt 18 V XR Brushless DCD791D2 2 × 2.0Ah Li-Ion, with empty battery. In the Virtual group, participants viewed a rendered 3D model (CAD model) of the same drill in a 3D viewer on a laptop. In the MR group, participants wore a Meta Quest 3 Virtual Reality (VR) headset, displaying a neutral virtual environment with a desk, similar to the room in which the experiment was conducted. Participants were handed the physical drill with a tracker attached to it, which mapped the real drill to the rendered 3D model of the drill in the virtual environment. To as high a degree as viable the prototypes across media were aligned, sharing form and aesthetic in all cases. In physical and MR this also extended to physicality, with each comprising a kinematically and geometrically accurate physical model of the drill. Differences then existed primarily in the way in which properties were presented to the user, i.e. Virtual and MR present aesthetic via a digital representation rather than physical. The only difference between digital aesthetic and physical drill was in their labels, with the physical marked as 18 V and digital marked as 20 V, due to CAD model availability. It is acknowledged that this alignment creates limitation in the potential that each media provides (i.e. a physical prototype would often ‘turn on’ and give functional feedback); close alignment of prototype behaviours across media is intended to add control, and closer align any cognitive differences to inherent differences between media rather than their functional capability.

**Fig. 6:**
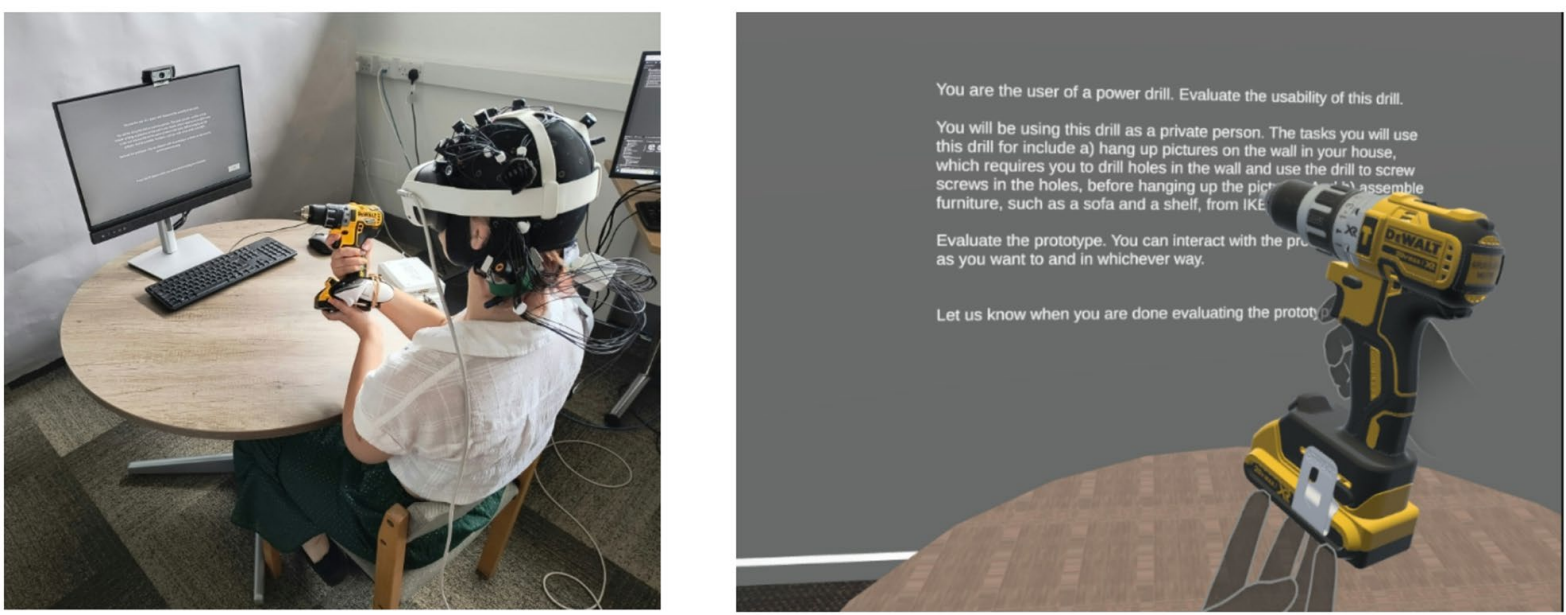
MR condition setup. Left) The real environment with user holding the drill. Right) Virtual environment from user’s perspective. The participant gave written informed consent to publication of these images in an online open access publication, in addition to the consent form for experiment participation.

**Fig. 7:**
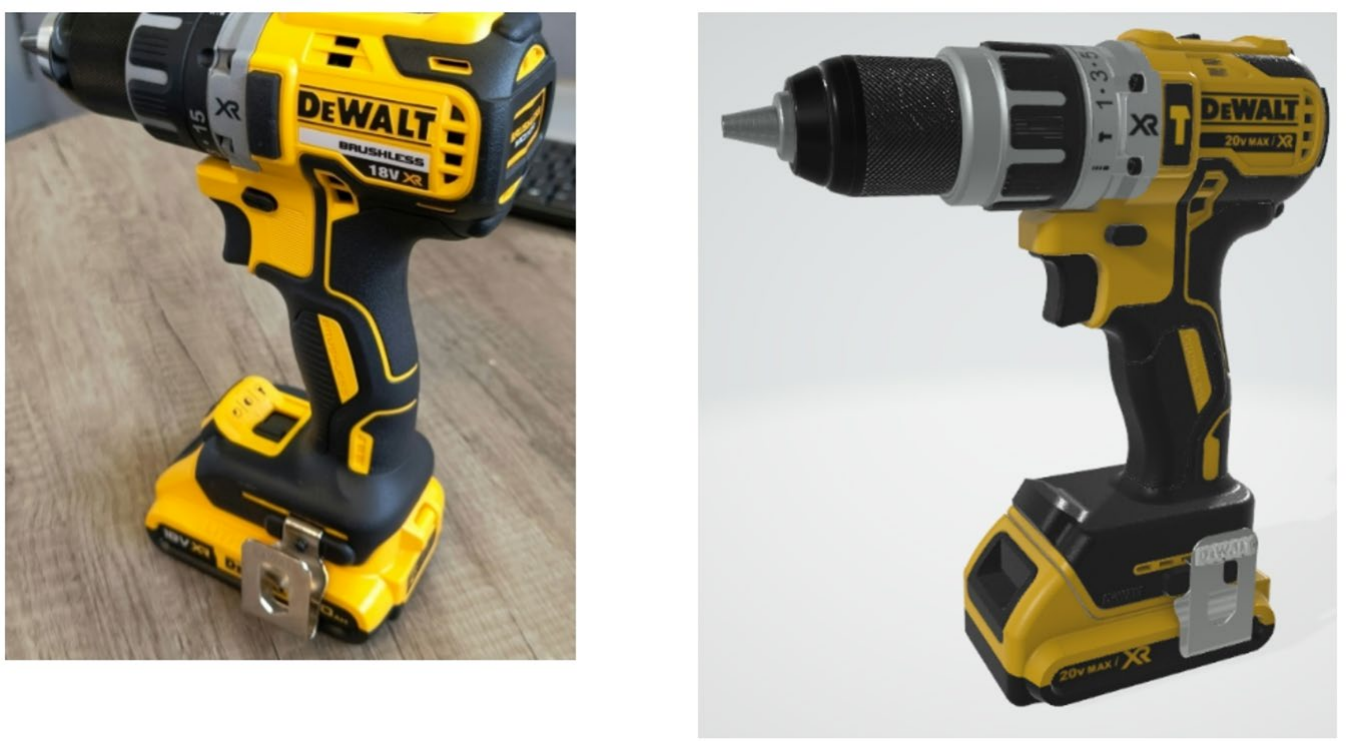
The physical and the virtual prototype.

**Technical description of MR scene setup and tracking of drill**: To create the MR environment, a Meta Quest 3 VR headset was used with a virtual environment created and managed through Unity (version 2022.3.23.f1). This headset was selected because it provides high quality visual rendering, reliable and accurate hand tracking (compared to other systems such as the LeapMotion IR tracking module) and a small form factor that ensured compatibility with the fNIRS sensors. This headset is not compatible with 3D trackers (e.g., HTC Vive trackers, which uses infrared light, which is incompatible with fNIRS sensors) commonly used to track physical objects position for insertion into virtual environments. A controller was therefore mounted on the physical drill using a custom mount (Fig. 6) to map the position of the virtually rendered drill to the same position as the physical drill relative to the participant’s perspective. Figure 7 depicts the physical and virtual drill. Participants were made aware of the existence and location of this tracker upon being handed the drill and asked to ignore its presence as much as possible.

The VR headset was wired to a computer managing the virtual environment using Unity version 2022.3.23.f1 (Unity Technologies, San Francisco, California). The Unity game engine has been adopted by other disciplines as it can manage and render complex 3D environments with realistic lighting and physical effects. We used Unity because it is effective for developing Virtual and MR environments for experimental purposes and prototyping^31,32^. The virtual environment’s position was mapped to the physical environment using “Spatial Anchors”, a feature of the Quest 3 headset that allows persistent 3D location markers to be inserted onto the headset’s model of the physical environment. The virtual environment was designed to be as similar as the physical environment as practicable (Fig. 6). To ensure the participant did not need to remove the Quest 3 headset during the experiment, the instructions for each task were rendered as text on the wall of the virtual environment. To interact with PsychoPy, participants were brought into Quest Link desktop environment in which there was a virtual desktop replicating the computer screen with PsychoPy on. The keyboard and mouse were moved within participants’ reach such that they could interact with PsychoPy in the same way as other groups. When conducting the experimental tasks, participants were brought into the Unity environment. Using these techniques, all visual information provided to participants in the MR condition was generated virtually, but the virtual–physical spatial mapping ensured drill had physicality and could be tangibly interacted with by the participant.

### Data collection

fNIRS data was collected throughout the experiment. Video recordings were made of the prototype evaluation task and design change task. The durations of both tasks were recorded. Participants answered a questionnaire after completing the tasks.

### fNIRS data collection

fNIRS data were collected with Aurora acquisition software using a continuous-wave NirSport2 device (NIRx Medical Technologies, LLC, Berlin, Germany) with 8 sources, 8 detectors and 8 short channels. LED sources emitted light at 760 nm and 850 nm, and data was collected at 10.17 Hz. Participants’ head circumference was measured and individually sized caps (Easycap GmbH, Wörthsee, Germany) set up for each participant.

**Fig. 8:**
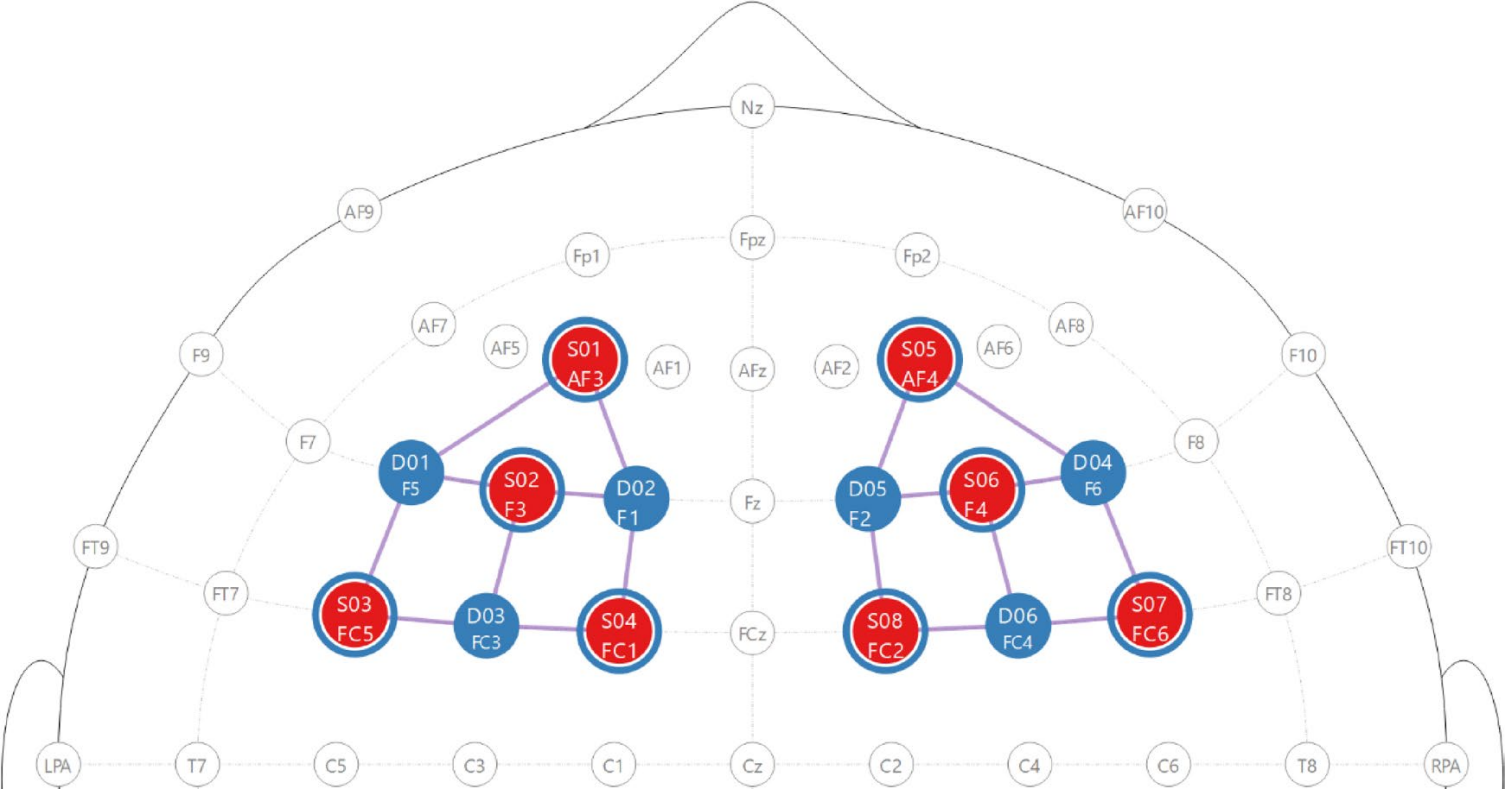
fNIRS montage. Red = light source, blue = detector, blue circle = short-channel measurement, purple line = measurement channel. Landmarks follows the 10 − 5 system nomenclature^34^.

The montage (Fig. 8) was designed to cover right and left dorsolateral prefrontal cortex (DLPFC) (Broadman area (BA) 9 and 46) while not interfering with the bands securing the VR headset to participants heads. Regions-of-interest (ROIs) were identified with fOLD v2.2^33^. Optodes were positioned according to the 10 − 5 system^34^. To be able to use the short-channels, the FC5 and FC6 source positions were added although its does not span the DLPFC, rather FC5-F5 and FC5-FC3 covers BA44.

#### Rationale for selected ROI

The frontal lobe is central in decision-making, it is involved in decision-making tasks ranging from binary decisions to multi-attribute decisions that require explicit deliberation and integration of different information sources^26^. The dorsolateral prefrontal cortex (DLPFC) is key for decisions that need to consider multiple information sources^26,35^. It is involved in manipulating decision relevant information online^26,36^ and in conscious deliberation during decisions^26^. Furthermore, the DLPFC is responsible for maintaining and manipulating information in working memory (WM). WM function is essential for maintaining focus on goal hierarchies, monitoring status of competing options, and possibly storing affective information related to options and attributes assessment, which has obvious value in decision-making^26^. The DLPFC is engaged when making intellectual effortful decisions^35^ and the categorization of novel stimuli, a process involving considerable comparison between competing options when making a decision. The DLPFC is also implicated in inductive reasoning, which sorts among competing arguments with gradations of utility providing answers that are more or less likely based on a body of evidence—it is similar to deciding amongst multiple options and novel categorization, as all are open-ended processes without objectively correct answers. Induction appears to activate certain areas of DLPFC predominantly, tending to be right lateralized within the frontal lobe^26^.

There appears to be a general trend toward a right hemispheric bias in processing novel information that requires a response that must be drawn from memory, whereas the left DLPFC seems key in producing responses to stimuli drawing directly upon environmental cues^26^. Furthermore, there exists hemispheric asymmetry in which open-ended, inductive processing is primarily right-lateralized in the DLPFC, ventrolateral prefrontal, and superior parietal regions^26^.

These cognitive functions are highly relevant for the tasks the participants were subjected to. The prototype evaluation task includes processing novel information, and is a multi-attribute decision making task requiring deliberation and integration of different information sources (visual, tactile, etc.). The design change task, where participants were asked to propose a design change, requires inductive reasoning and is an open-ended process without objectively correct answers. Both tasks could, depending on individual participants and their cognitive effort, be intellectually effortful decision-making processes. We have therefore selected the DLPFC as the focal region for this research.

### Questionnaire: subjective data collection

In addition to workload, affective state, and stress, the questionnaire contained questions about the product and the technology used to present the prototype. It is not within the scope of this article to analyse the product and technology questions. All questions are included in Supplementary Materials.

**Workload**,** affective state and stress** were collected with NASA Task Load Index (NASA-TLX) dimensions, without the weighting process, i.e., Raw TLX^23,37^, and Overall workload^24^ assessed workload. Arousal and Valence from the Affect grid^25^ assessed affective (mental) state. We also recorded participants’ stress level.

**Product questions**, i.e., questions about the design of the prototype, were based on Houde & Hill’s^2^ dimensions: Role, Look and Feel, and Implementation. We formed two questions for each dimension, together addressing to which degree the prototype was appropriately design for the task. The questions concerned: Role: performance of drill to specified task, using drill with appropriate precision; Look and Feel: appropriate drill mass, appropriate drill size; and Implementation: intuitiveness of interface, ease of changing battery. Participants also rated their confidence in their answers.

**Technology questions**, i.e., questions about the technology used to present the prototype (i.e., physical, virtual, and MR), constituted most of the questionnaire. An adaptation of the System Usability Scale (SUS)^38^ was used to assess usability. To assess affordances we used two dimensions of value for MR prototypes^10^, as only two were appropriate for this experimental context (visualization and knowledge management), and a select subset of affordances for MR prototypes^9^, appropriate for this experimental context (flexibility, fidelity, analytic capacity, stakeholder accessibility, breath of learning, interactivity, and feedback immediacy).

Questions were phrased as Likert-scale statements to which participants rated their level of agreement with, ranging from “Strongly disagree” to “Strongly agree” on 5-point scale (SUS), 7-point scale (product questions, dimensions of value, affordances) and 11-point scale (NASA-TLX, Overall workload, arousal, valence, stress).

Collected demographics included age, biological sex, handedness, student (Yes/No), design experience (years), caffeine intake, participants experience with VR, CAD, physical prototyping, and with using a real power drill. Caffeine intake was recorded, but not controlled.

### Participants

Participants were recruited through posters around the University of Bristol campus, internal communication channels, and social media. Participants were screened prior to participation, and only participated if they fit the following inclusion criteria:

- Age above 18 years old.
- Understand written and spoken English.
- Do not have any neurological or psychiatric conditions (e.g., ADHD, autism, epilepsy).
- Do not take any prescribed medication that could affect brain function (e.g., stimulants, antidepressants, sleeping medication).
- Do not have a history of alcohol or drug abuse that could affect typical brain function.

Participant sample: Based on the conventional sample size rule of thumb of 30 participants per group when measuring group differences, we aimed to recruit a minimum of 30 participants per group. 103 healthy adults participated in the experiment. 15 participants were discarded due to incompliance with the procedure or technical errors, leaving *N* = 88 participants for the final sample. See Table 2 for descriptive statistics.

Ethical approval was obtained from The Faculty of Engineering Research Ethics Committee at University of Bristol (Ref: 18404). Participants gave written informed consent before participation. The experiment was conducted in accordance with the Declaration of Helsinki.

**Table 2:** Descriptive statistics of participant demographics.

	Group	Virtual	Physical	MR
	N	30	30	28
Female	N	11	10	10
	%	36.7	33.3	35.7
Male	N	19	20	18
	%	63.3	66.7	64.3
Age (years)	M	32.07	26.9	32.32
	SD	12.77	7.49	8.79
Handedness	Right	27	29	24
	Left	2	1	3
	Ambi-dextrous	1	0	1
Student (yes)	N	15	20	10
	%	50	66.7	35.7
VR experience^1^	M	3.33	3.93	4.29
	SD	2.07	2.84	2.83
CAD experience^1^	M	5.2	5.4	4.39
	SD	3.49	3.28	3.41
Physical prototyping experience^1^	M	4.57	4.97	4
	SD	3.53	3.42	3.22
Real drill experience^1^	M	7.1	6.77	6.54
	SD	3	2.86	3.32
Design/engineering experience (years)	M	5.77	3.07	3.5
	SD	9.24	4.04	5.12

^1^Measured on a 1–11-point scale.

### Data analysis

#### Between-group differences on individual demographic variables

To ensure the groups were balanced, we investigated whether there were any between-group differences in demographic variables. These analyses were undertaken in RStudio 2023.12.1^39^ using R version 4.3.3^40^. We ran a chi-squared test [chisq.test^40^ to assess whether there were differences in biological sex and the number of student participants between groups. For post-hoc pairwise comparisons we ran pairwise chi-squared tests with a Bonferroni correction [stats::p.adjust^40^. To assess whether there were differences in handedness between groups we ran a Fisher’s exact test [stats::fisher.test^40^, because some contingency table frequencies were below 5. For post-hoc comparisons we ran pairwise Fisher’s exact test with a Bonferroni correction [stats::p.adjust^40^. Statistical significance is ascertained if *p* < 0.05. For continuous and ordinal demographical variables (age, design experience, VR experience, CAD experience, experience with physical prototyping, and experience with using a real drill) we ran a general linear model (GLM) [lm^40^] and inspected GLM assumptions (normality of residuals, homoscedasticity, and influential cases) visually ([ggplot2::autoplot^41^ produced Residuals vs. Fitted-, Scale-Location-, Normal Q-Q-, and Cook’s distance plots). All variables violated one or more of the assumptions, and we therefore ran a robust model instead. We opted for a bootstrap version of the heteroscedastic one-way ANOVA for trimmed means [WRS2::t1waybt^42^ predicting each demographic variable from group, using a 20% trim and 1000 bootstrap samples, and corresponding post-hoc tests [WRS2::mcppb20^42^] which uses Hochberg’s 1988 sharper Bonferroni procedure to control family-wise error rate. We report the test statistic ($\psi$) and 95% confidence intervals. Statistical significance is ascertained if confidence intervals do not cross zero.

#### Between-group differences on workload, affective state, and stress

These are ordinal variables, so we used the same approach as described above, i.e., either GLM or a robust model. GLMs were used for arousal, valence, performance. GLM post-hoc comparisons were computed using a Bonferroni correction [modelbased::estimate_contrasts^43^. Statistical significance is ascertained if *p* < 0.05. Robust models were used for mental demand, temporal demand, effort, frustration, overall workload, and stress. For physical demand we also conducted the Kruskal–Wallis rank sum Test [stats::kruskal.test^40^ and post-hoc pairwise comparisons with the Dunn test [FSA::dunnTest^44^ with a Holm correction^45^. See Supplementary Materials for further details on physical demand. Statistical significance is ascertained if *p* < 0.05. Epsilon squared [rcompanion::epsilonSquared^46^ estimated effect size. We report the Z-value, p-value, and effect size.

#### fNIRS data analysis

fNIRS data were analysed with NIRS Brain AnalyzIR Toolbox^47^ in MATLAB R2023b (The MathWorks Inc., Natick, Massachusetts). Raw data was trimmed to the start and end of the 1-back task for it to serve its purpose as baseline. Raw light intensities were converted to optical density and signal quality check conducted on optical density data. For signal quality assessment we used QT-NIRS^48,49^ with default thresholds: Scalp Coupling Index (SCI) = 0.8, Peak Spectral Power (PSP) = 0.1, 75% overall data quality, calculated per channel with a 5-second time windows. Data was discarded automatically on a per-channel basis if it did not meet these criteria. Data meeting these criteria were labelled high-quality. Overall, 72.77% of the data were high quality (for physical group (*N* = 30): 71.03%, virtual group (*N* = 30): 73.72%, MR group (*N* = 28): 73.63%), and thus retained for subsequent analysis. Pruned optical density data was converted to oxygenated (HbO) and deoxygenated (HbR) haemoglobin data using the modified Beer-Lambert Law^50^, with a partial pathlength factor of 0.1^51^.

**Between-group analysis**: We created three separate analysis models to investigate between-group differences in haemodynamic activation. These models included (1) both tasks, (2) evaluation task only, and (3) design change task only, aiming to answer the following questions.

1. GLM1: Is there a between-group difference in activation overall (i.e., including both tasks)?
2. GLM2: Is there a between-group difference in activation during evaluation only?
3. GLM3: Is there a between-group difference in activation during design change only?

The same pipeline, described in the following, was used for all three analyses, but rerun as tasks and baselines were different. One participant had erroneous triggers for the evaluation task, leaving 87 participants for the evaluation model (GLM2). For 1st (i.e., participant) level statistics, pruned haemoglobin data were submitted to a general linear model (GLM) with a canonical haemodynamic response function that used the AR-IRLS algorithm^52,53^, with added short-channel regressors, to obtain estimates of regression coefficients (i.e., beta values representing HbO/HbR activation per condition in contrast to baseline, per participant). Statistical leverage for a group model was calculated per participant. For all three analyses no participant contributed significant leverage and thus all participants were retained for the group model. 1st level statistics were submitted to a 2nd (i.e., group) level model using a robust mixed-effects model that included main effect of group, controlling for participant and age as random variables. These results were used for group-level contrasts (t-tests). To investigate between-group differences we set up contrasts reflecting all pairwise comparisons of groups, i.e., Physical-Virtual, Physical-MR, and MR-Virtual. The Benjamini-Hochberg procedure was used for false discovery (FDR) correction, the corrected p-value denoted as q^54^. Results are presented as t-statistical maps plotted according to the 10 − 5 system^34^.

**Main effect of age analysis**: Because there was a significant difference in age between Physical and MR we ran additional fNIRS analyses to investigate whether there was a significant main effect of age on fNIRS activation. The preprocessing pipeline was the same as described previously. The 2nd -level group model included main effect of age, controlling for participant as a random variable. Because there was a significant main effect of age on one HbO channel, GLM1-3 was changed to include age as a random variable and reran.

## Supplementary Information

Below is the link to the electronic supplementary material.

Supplementary Material 1

## Data Availability

The datasets generated during and/or analysed during the current study are available in the Open Science Framework repository: “The 21st Century Prototyping Affordance study”. https://doi.org/10.17605/OSF.IO/XH9YW.
