# Supplementary material for: Users evaluating physical, virtual, and mixed reality prototypes exhibit differential DLPFC brain activity
Source: Sci Rep. 2025 Nov 12;15:39729. doi: 10.1038/s41598-025-23557-z (PMC12612208; doi:10.1038/s41598-025-23557-z)
Supplement: Supplementary file 1 — Supplementary Material 1 [file 41598_2025_23557_MOESM1_ESM.docx]

Supplementary Materials

# Task text

**Evaluation task:**

You are the user of a power drill. Evaluate the usability of this drill.

You will be using this drill as a private person. The tasks you will use this drill for include a) hang up pictures on the wall in your house, which requires you to drill holes in the wall and use the drill to screw screws in the holes, before hanging up the pictures. And b) assemble furniture, such as a sofa and a shelf, from IKEA.

Evaluate the prototype. You can interact with the prototype as much as you want to and in whichever way.

Press the OK button when you are done evaluating the prototype.

**Design change task:**

What would you change on the current drill design?

Articulate (explain by speaking out loud) one change you would want to make to the drill.

You can still interact with the prototype as much as you want to.

Press the OK button when you are done.

# Statistical tables for fNIRS results

## Main effect of age

Table 1 HbO activation results for robust mixed effects model including main effect of age, controlling for subject. Significant channels (q<0.05) are shown as sold lines. The t-statistic is scaled to [-5, 5]. Channels are displayed on top of the 10-20 coordinate system. HbR activation results are mot depcited as there were no sigificant channels.


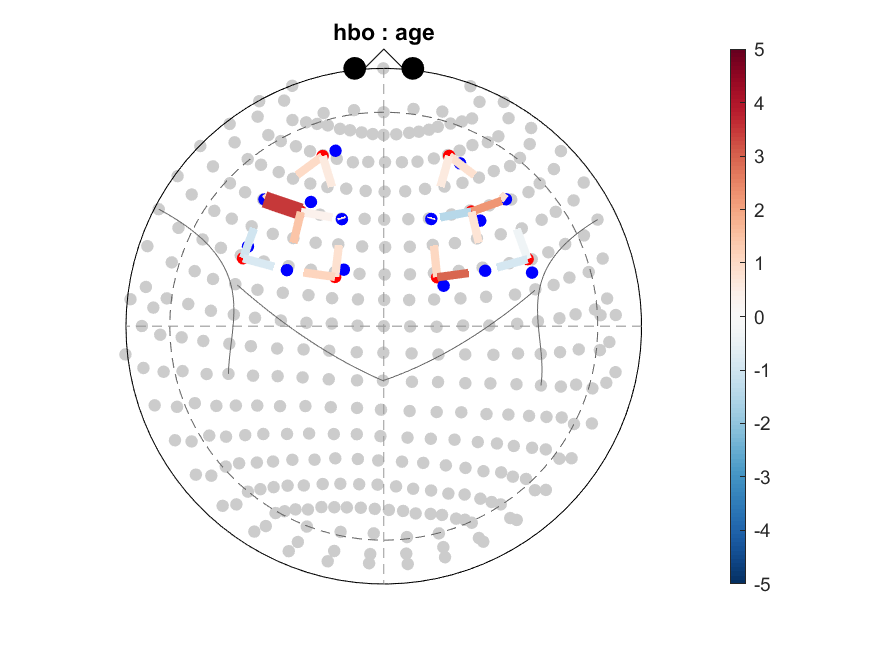


Table 2 Results: 2^nd^ level group statistics main effects of age. Significant channels (q<0.05) are highlighted in green with an asterisk (*).

| source | detector | type | beta | se | tstat | dfe | q | Min  Discoverable  Change | Relative  Power |
| --- | --- | --- | --- | --- | --- | --- | --- | --- | --- |
| 1 | 1 | hbo | 0.1 | 0.102 | 0.9779 | 174 | 0.6469 | 0.255 | 0.379 |
| 1 | 1 | hbr | -0.079 | 0.07 | -1.136 | 174 | 0.6299 | 0.174 | 0.555 |
| 1 | 2 | hbo | 0.043 | 0.07 | 0.6154 | 174 | 0.6469 | 0.175 | 0.553 |
| 1 | 2 | hbr | -0.07 | 0.043 | -1.638 | 174 | 0.372 | 0.106 | 0.909 |
| 2 | 1 | hbo | 0.281 | 0.08 | 3.5251 | 174 | 0.0195* | 0.199 | 0.485 |
| 2 | 1 | hbr | -0.03 | 0.046 | -0.638 | 174 | 0.6469 | 0.116 | 0.835 |
| 2 | 2 | hbo | 0.019 | 0.056 | 0.3428 | 174 | 0.7753 | 0.14 | 0.691 |
| 2 | 2 | hbr | -0.084 | 0.039 | -2.165 | 174 | 0.2283 | 0.097 | 1 |
| 2 | 3 | hbo | 0.123 | 0.085 | 1.4379 | 174 | 0.4568 | 0.213 | 0.453 |
| 2 | 3 | hbr | 0.019 | 0.054 | 0.344 | 174 | 0.7753 | 0.135 | 0.713 |
| 3 | 1 | hbo | -0.078 | 0.077 | -1.008 | 174 | 0.6469 | 0.192 | 0.503 |
| 3 | 1 | hbr | -0.081 | 0.043 | -1.879 | 174 | 0.2786 | 0.108 | 0.897 |
| 3 | 3 | hbo | -0.068 | 0.08 | -0.845 | 174 | 0.6469 | 0.2 | 0.483 |
| 3 | 3 | hbr | -0.039 | 0.057 | -0.684 | 174 | 0.6469 | 0.144 | 0.673 |
| 4 | 2 | hbo | 0.063 | 0.075 | 0.8343 | 174 | 0.6469 | 0.188 | 0.513 |
| 4 | 2 | hbr | -0.09 | 0.052 | -1.739 | 174 | 0.3352 | 0.13 | 0.745 |
| 4 | 3 | hbo | 0.087 | 0.078 | 1.11 | 174 | 0.6299 | 0.195 | 0.496 |
| 4 | 3 | hbr | -0.065 | 0.053 | -1.209 | 174 | 0.6299 | 0.134 | 0.723 |
| 5 | 4 | hbo | 0.149 | 0.187 | 0.7972 | 174 | 0.6469 | 0.467 | 0.207 |
| 5 | 4 | hbr | 0.021 | 0.091 | 0.2311 | 174 | 0.8175 | 0.228 | 0.423 |
| 5 | 5 | hbo | 0.033 | 0.052 | 0.6272 | 174 | 0.6469 | 0.131 | 0.739 |
| 5 | 5 | hbr | -0.017 | 0.039 | -0.436 | 174 | 0.7706 | 0.097 | 0.991 |
| 6 | 4 | hbo | 0.237 | 0.104 | 2.2701 | 174 | 0.2199 | 0.261 | 0.371 |
| 6 | 4 | hbr | -0.124 | 0.063 | -1.955 | 174 | 0.2686 | 0.158 | 0.61 |
| 6 | 5 | hbo | -0.091 | 0.062 | -1.469 | 174 | 0.4568 | 0.154 | 0.625 |
| 6 | 5 | hbr | -0.078 | 0.039 | -2.016 | 174 | 0.2686 | 0.097 | 1 |
| 6 | 6 | hbo | 0.09 | 0.12 | 0.7468 | 174 | 0.6469 | 0.301 | 0.321 |
| 6 | 6 | hbr | -0.06 | 0.074 | -0.806 | 174 | 0.6469 | 0.185 | 0.523 |
| 7 | 4 | hbo | -0.044 | 0.115 | -0.386 | 174 | 0.7753 | 0.287 | 0.337 |
| 7 | 4 | hbr | -0.197 | 0.074 | -2.675 | 174 | 0.0982 | 0.184 | 0.526 |
| 7 | 6 | hbo | -0.071 | 0.08 | -0.879 | 174 | 0.6469 | 0.201 | 0.481 |
| 7 | 6 | hbr | 0.012 | 0.049 | 0.2451 | 174 | 0.8175 | 0.123 | 0.784 |
| 8 | 5 | hbo | 0.079 | 0.073 | 1.0837 | 174 | 0.6299 | 0.183 | 0.528 |
| 8 | 5 | hbr | -0.033 | 0.049 | -0.671 | 174 | 0.6469 | 0.123 | 0.785 |
| 8 | 6 | hbo | 0.293 | 0.099 | 2.9728 | 174 | 0.0607 | 0.246 | 0.393 |
| 8 | 6 | hbr | -0.043 | 0.063 | -0.68 | 174 | 0.6469 | 0.157 | 0.614 |

## Glm1: Between-group differences in overall activation (i.e., including both tasks)

Table 3 Contrast statistics for between group-differences in overall activation, i.e., including both tasks. Significant channels (q<0.05) are highlighted in green with an asterisk (*).

| source | detector | type | contrast | beta | se | t-stat | dfe | q | Min  Discoverable  Change | Relative  Power |
| --- | --- | --- | --- | --- | --- | --- | --- | --- | --- | --- |
| 1 | 1 | hbo | Physical-Virtual | -2.375 | 3.066 | -0.775 | 172 | 0.8155 | 7.656 | 0.209 |
| 1 | 1 | hbo | Physical-MR | -0.993 | 2.834 | -0.35 | 172 | 0.8732 | 7.078 | 0.226 |
| 1 | 1 | hbo | MR-Virtual | -1.382 | 2.619 | -0.528 | 172 | 0.8732 | 6.541 | 0.245 |
| 1 | 1 | hbr | Physical-Virtual | -0.124 | 1.819 | -0.068 | 172 | 0.9726 | 4.544 | 0.353 |
| 1 | 1 | hbr | Physical-MR | -2.131 | 1.594 | -1.337 | 172 | 0.659 | 3.981 | 0.403 |
| 1 | 1 | hbr | MR-Virtual | 2.0062 | 1.684 | 1.1915 | 172 | 0.6682 | 4.205 | 0.381 |
| 1 | 2 | hbo | Physical-Virtual | -1.98 | 2.163 | -0.915 | 172 | 0.7654 | 5.403 | 0.297 |
| 1 | 2 | hbo | Physical-MR | 0.1644 | 1.856 | 0.0886 | 172 | 0.9726 | 4.635 | 0.346 |
| 1 | 2 | hbo | MR-Virtual | -2.144 | 2.038 | -1.052 | 172 | 0.7145 | 5.09 | 0.315 |
| 1 | 2 | hbr | Physical-Virtual | 0.1951 | 1.307 | 0.1493 | 172 | 0.952 | 3.263 | 0.491 |
| 1 | 2 | hbr | Physical-MR | -2.103 | 1.17 | -1.797 | 172 | 0.4574 | 2.923 | 0.548 |
| 1 | 2 | hbr | MR-Virtual | 2.2986 | 1.164 | 1.9751 | 172 | 0.3365 | 2.907 | 0.551 |
| 2 | 1 | hbo | Physical-Virtual | 0.787 | 1.794 | 0.4388 | 172 | 0.8732 | 4.48 | 0.358 |
| 2 | 1 | hbo | Physical-MR | 1.4682 | 1.878 | 0.7819 | 172 | 0.8155 | 4.69 | 0.342 |
| 2 | 1 | hbo | MR-Virtual | -0.681 | 1.741 | -0.391 | 172 | 0.8732 | 4.349 | 0.369 |
| 2 | 1 | hbr | Physical-Virtual | -0.383 | 0.853 | -0.449 | 172 | 0.8732 | 2.13 | 0.753 |
| 2 | 1 | hbr | Physical-MR | -0.275 | 0.853 | -0.322 | 172 | 0.8732 | 2.131 | 0.752 |
| 2 | 1 | hbr | MR-Virtual | -0.108 | 0.864 | -0.125 | 172 | 0.9628 | 2.158 | 0.743 |
| 2 | 2 | hbo | Physical-Virtual | -3.622 | 1.108 | -3.27 | 172 | 0.0351* | 2.767 | 0.579 |
| 2 | 2 | hbo | Physical-MR | -4.145 | 1.358 | -3.052 | 172 | 0.0407* | 3.393 | 0.472 |
| 2 | 2 | hbo | MR-Virtual | 0.5232 | 1.428 | 0.3664 | 172 | 0.8732 | 3.567 | 0.449 |
| 2 | 2 | hbr | Physical-Virtual | 0.6345 | 0.733 | 0.8659 | 172 | 0.7742 | 1.83 | 0.876 |
| 2 | 2 | hbr | Physical-MR | 0.5718 | 0.897 | 0.6376 | 172 | 0.836 | 2.24 | 0.716 |
| 2 | 2 | hbr | MR-Virtual | 0.0627 | 0.642 | 0.0978 | 172 | 0.9726 | 1.603 | 1 |
| 2 | 3 | hbo | Physical-Virtual | -1.903 | 2.608 | -0.73 | 172 | 0.8261 | 6.514 | 0.246 |
| 2 | 3 | hbo | Physical-MR | -2.262 | 2.455 | -0.921 | 172 | 0.7654 | 6.132 | 0.261 |
| 2 | 3 | hbo | MR-Virtual | 0.3592 | 2.352 | 0.1527 | 172 | 0.952 | 5.874 | 0.273 |
| 2 | 3 | hbr | Physical-Virtual | 0.9906 | 1.352 | 0.7326 | 172 | 0.8261 | 3.377 | 0.475 |
| 2 | 3 | hbr | Physical-MR | -0.313 | 1.301 | -0.241 | 172 | 0.8928 | 3.249 | 0.493 |
| 2 | 3 | hbr | MR-Virtual | 1.3036 | 1.312 | 0.9933 | 172 | 0.7558 | 3.278 | 0.489 |
| 3 | 1 | hbo | Physical-Virtual | 6.5378 | 2.135 | 3.0616 | 172 | 0.0407* | 5.333 | 0.301 |
| 3 | 1 | hbo | Physical-MR | 4.4485 | 2.131 | 2.0879 | 172 | 0.2953 | 5.321 | 0.301 |
| 3 | 1 | hbo | MR-Virtual | 2.0893 | 2 | 1.0446 | 172 | 0.7145 | 4.996 | 0.321 |
| 3 | 1 | hbr | Physical-Virtual | 3.3671 | 1.063 | 3.1672 | 172 | 0.0393* | 2.655 | 0.604 |
| 3 | 1 | hbr | Physical-MR | 2.032 | 0.962 | 2.1125 | 172 | 0.2953 | 2.402 | 0.667 |
| 3 | 1 | hbr | MR-Virtual | 1.335 | 0.938 | 1.4229 | 172 | 0.6515 | 2.343 | 0.684 |
| 3 | 3 | hbo | Physical-Virtual | -1.595 | 2.308 | -0.691 | 172 | 0.836 | 5.765 | 0.278 |
| 3 | 3 | hbo | Physical-MR | -0.066 | 2.071 | -0.032 | 172 | 0.9838 | 5.172 | 0.31 |
| 3 | 3 | hbo | MR-Virtual | -1.529 | 2.195 | -0.697 | 172 | 0.836 | 5.483 | 0.292 |
| 3 | 3 | hbr | Physical-Virtual | 0.6533 | 1.458 | 0.4482 | 172 | 0.8732 | 3.641 | 0.44 |
| 3 | 3 | hbr | Physical-MR | 2.2528 | 1.348 | 1.6708 | 172 | 0.4838 | 3.367 | 0.476 |
| 3 | 3 | hbr | MR-Virtual | -1.599 | 1.377 | -1.162 | 172 | 0.6838 | 3.439 | 0.466 |
| 4 | 2 | hbo | Physical-Virtual | -2.477 | 2.259 | -1.097 | 172 | 0.7054 | 5.642 | 0.284 |
| 4 | 2 | hbo | Physical-MR | -0.776 | 2.228 | -0.348 | 172 | 0.8732 | 5.565 | 0.288 |
| 4 | 2 | hbo | MR-Virtual | -1.701 | 1.832 | -0.929 | 172 | 0.7654 | 4.575 | 0.35 |
| 4 | 2 | hbr | Physical-Virtual | 1.6196 | 1.35 | 1.2001 | 172 | 0.6682 | 3.371 | 0.476 |
| 4 | 2 | hbr | Physical-MR | 0.0721 | 1.331 | 0.0542 | 172 | 0.9749 | 3.324 | 0.482 |
| 4 | 2 | hbr | MR-Virtual | 1.5475 | 1.106 | 1.3991 | 172 | 0.6544 | 2.763 | 0.58 |
| 4 | 3 | hbo | Physical-Virtual | -0.832 | 2.278 | -0.365 | 172 | 0.8732 | 5.69 | 0.282 |
| 4 | 3 | hbo | Physical-MR | -2.557 | 2.135 | -1.197 | 172 | 0.6682 | 5.333 | 0.301 |
| 4 | 3 | hbo | MR-Virtual | 1.725 | 2.256 | 0.7647 | 172 | 0.8155 | 5.634 | 0.285 |
| 4 | 3 | hbr | Physical-Virtual | 2.1232 | 1.29 | 1.6454 | 172 | 0.4838 | 3.223 | 0.497 |
| 4 | 3 | hbr | Physical-MR | -0.099 | 1.253 | -0.079 | 172 | 0.9726 | 3.13 | 0.512 |
| 4 | 3 | hbr | MR-Virtual | 2.2224 | 1.246 | 1.7837 | 172 | 0.4574 | 3.112 | 0.515 |
| 5 | 4 | hbo | Physical-Virtual | 3.051 | 5.233 | 0.5831 | 172 | 0.8732 | 13.07 | 0.123 |
| 5 | 4 | hbo | Physical-MR | -1.895 | 4.574 | -0.414 | 172 | 0.8732 | 11.42 | 0.14 |
| 5 | 4 | hbo | MR-Virtual | 4.9462 | 4.706 | 1.051 | 172 | 0.7145 | 11.75 | 0.136 |
| 5 | 4 | hbr | Physical-Virtual | 1.3882 | 2.79 | 0.4975 | 172 | 0.8732 | 6.969 | 0.23 |
| 5 | 4 | hbr | Physical-MR | -2.736 | 2.149 | -1.273 | 172 | 0.6682 | 5.368 | 0.299 |
| 5 | 4 | hbr | MR-Virtual | 4.124 | 2.516 | 1.6391 | 172 | 0.4838 | 6.284 | 0.255 |
| 5 | 5 | hbo | Physical-Virtual | 0.4374 | 1.663 | 0.2631 | 172 | 0.8839 | 4.153 | 0.386 |
| 5 | 5 | hbo | Physical-MR | 1.205 | 1.558 | 0.7732 | 172 | 0.8155 | 3.892 | 0.412 |
| 5 | 5 | hbo | MR-Virtual | -0.768 | 1.584 | -0.485 | 172 | 0.8732 | 3.956 | 0.405 |
| 5 | 5 | hbr | Physical-Virtual | 0.7612 | 0.871 | 0.8742 | 172 | 0.7742 | 2.175 | 0.737 |
| 5 | 5 | hbr | Physical-MR | -0.236 | 0.901 | -0.262 | 172 | 0.8839 | 2.249 | 0.713 |
| 5 | 5 | hbr | MR-Virtual | 0.9968 | 1.026 | 0.9711 | 172 | 0.7648 | 2.564 | 0.625 |
| 6 | 4 | hbo | Physical-Virtual | 0.782 | 2.611 | 0.2996 | 172 | 0.8788 | 6.52 | 0.246 |
| 6 | 4 | hbo | Physical-MR | 0.0178 | 2.356 | 0.0076 | 172 | 0.994 | 5.884 | 0.272 |
| 6 | 4 | hbo | MR-Virtual | 0.7642 | 2.414 | 0.3166 | 172 | 0.8732 | 6.029 | 0.266 |
| 6 | 4 | hbr | Physical-Virtual | -0.653 | 1.318 | -0.496 | 172 | 0.8732 | 3.291 | 0.487 |
| 6 | 4 | hbr | Physical-MR | -1.399 | 1.268 | -1.104 | 172 | 0.7054 | 3.167 | 0.506 |
| 6 | 4 | hbr | MR-Virtual | 0.746 | 1.309 | 0.5698 | 172 | 0.8732 | 3.27 | 0.49 |
| 6 | 5 | hbo | Physical-Virtual | 3.7357 | 1.584 | 2.3586 | 172 | 0.23 | 3.956 | 0.405 |
| 6 | 5 | hbo | Physical-MR | 0.7961 | 1.859 | 0.4282 | 172 | 0.8732 | 4.644 | 0.345 |
| 6 | 5 | hbo | MR-Virtual | 2.9396 | 1.754 | 1.6759 | 172 | 0.4838 | 4.381 | 0.366 |
| 6 | 5 | hbr | Physical-Virtual | 0.504 | 0.784 | 0.6425 | 172 | 0.836 | 1.959 | 0.818 |
| 6 | 5 | hbr | Physical-MR | -0.323 | 0.914 | -0.353 | 172 | 0.8732 | 2.284 | 0.702 |
| 6 | 5 | hbr | MR-Virtual | 0.8269 | 0.892 | 0.9273 | 172 | 0.7654 | 2.227 | 0.72 |
| 6 | 6 | hbo | Physical-Virtual | 3.5889 | 3.238 | 1.1085 | 172 | 0.7054 | 8.086 | 0.198 |
| 6 | 6 | hbo | Physical-MR | -3.071 | 3.578 | -0.858 | 172 | 0.7742 | 8.936 | 0.179 |
| 6 | 6 | hbo | MR-Virtual | 6.6604 | 3.02 | 2.2052 | 172 | 0.2824 | 7.544 | 0.213 |
| 6 | 6 | hbr | Physical-Virtual | -5.856 | 1.583 | -3.699 | 172 | 0.0109* | 3.954 | 0.405 |
| 6 | 6 | hbr | Physical-MR | -2.354 | 1.848 | -1.274 | 172 | 0.6682 | 4.616 | 0.347 |
| 6 | 6 | hbr | MR-Virtual | -3.502 | 1.67 | -2.098 | 172 | 0.2953 | 4.17 | 0.384 |
| 7 | 4 | hbo | Physical-Virtual | 9.2664 | 2.512 | 3.6888 | 172 | 0.0109* | 6.274 | 0.256 |
| 7 | 4 | hbo | Physical-MR | 10.092 | 2.533 | 3.9838 | 172 | 0.0108* | 6.327 | 0.253 |
| 7 | 4 | hbo | MR-Virtual | -0.826 | 2.463 | -0.335 | 172 | 0.8732 | 6.153 | 0.261 |
| 7 | 4 | hbr | Physical-Virtual | -0.658 | 1.391 | -0.473 | 172 | 0.8732 | 3.474 | 0.461 |
| 7 | 4 | hbr | Physical-MR | 0.8289 | 1.499 | 0.553 | 172 | 0.8732 | 3.744 | 0.428 |
| 7 | 4 | hbr | MR-Virtual | -1.487 | 1.236 | -1.202 | 172 | 0.6682 | 3.088 | 0.519 |
| 7 | 6 | hbo | Physical-Virtual | 4.3692 | 2.598 | 1.682 | 172 | 0.4838 | 6.488 | 0.247 |
| 7 | 6 | hbo | Physical-MR | 6.0025 | 2.583 | 2.324 | 172 | 0.23 | 6.451 | 0.248 |
| 7 | 6 | hbo | MR-Virtual | -1.633 | 2.514 | -0.65 | 172 | 0.836 | 6.28 | 0.255 |
| 7 | 6 | hbr | Physical-Virtual | 0.8817 | 1.389 | 0.6348 | 172 | 0.836 | 3.469 | 0.462 |
| 7 | 6 | hbr | Physical-MR | 2.9493 | 1.465 | 2.0129 | 172 | 0.3289 | 3.659 | 0.438 |
| 7 | 6 | hbr | MR-Virtual | -2.068 | 1.454 | -1.422 | 172 | 0.6515 | 3.632 | 0.441 |
| 8 | 5 | hbo | Physical-Virtual | -3.881 | 2.88 | -1.348 | 172 | 0.659 | 7.193 | 0.223 |
| 8 | 5 | hbo | Physical-MR | 1.4891 | 2.727 | 0.546 | 172 | 0.8732 | 6.812 | 0.235 |
| 8 | 5 | hbo | MR-Virtual | -5.37 | 2.3 | -2.335 | 172 | 0.23 | 5.743 | 0.279 |
| 8 | 5 | hbr | Physical-Virtual | 0.49 | 1.407 | 0.3483 | 172 | 0.8732 | 3.514 | 0.456 |
| 8 | 5 | hbr | Physical-MR | -0.957 | 1.449 | -0.66 | 172 | 0.836 | 3.62 | 0.443 |
| 8 | 5 | hbr | MR-Virtual | 1.4468 | 1.192 | 1.2135 | 172 | 0.6682 | 2.978 | 0.538 |
| 8 | 6 | hbo | Physical-Virtual | -4.307 | 3.213 | -1.341 | 172 | 0.659 | 8.025 | 0.2 |
| 8 | 6 | hbo | Physical-MR | -2.825 | 3.308 | -0.854 | 172 | 0.7742 | 8.261 | 0.194 |
| 8 | 6 | hbo | MR-Virtual | -1.482 | 3.244 | -0.457 | 172 | 0.8732 | 8.101 | 0.198 |
| 8 | 6 | hbr | Physical-Virtual | 2.5174 | 1.626 | 1.5484 | 172 | 0.5551 | 4.061 | 0.395 |
| 8 | 6 | hbr | Physical-MR | 0.4276 | 1.599 | 0.2674 | 172 | 0.8839 | 3.994 | 0.401 |
| 8 | 6 | hbr | MR-Virtual | 2.0898 | 1.661 | 1.2585 | 172 | 0.6682 | 4.148 | 0.386 |

## Glm2: Between-group differences in activation during evaluation task

Table 4 Contrast statistics for between group-differences in activation during evaluation task. Significant channels (q<0.05) are highlighted in green with an asterisk (*).

| source | detector | type | contrast | beta | se | t-stat | dfe | q | Min  Discoverable  Change | Relative  Power |
| --- | --- | --- | --- | --- | --- | --- | --- | --- | --- | --- |
| 1 | 1 | hbo | Physical-Virtual | -0.207 | 3.234 | -0.064 | 84 | 0.958 | 8.116 | 0.393 |
| 1 | 1 | hbo | Physical-MR | -1.7 | 3.15 | -0.54 | 84 | 0.8299 | 7.906 | 0.404 |
| 1 | 1 | hbo | MR-Virtual | 1.493 | 2.91 | 0.513 | 84 | 0.8299 | 7.302 | 0.437 |
| 1 | 1 | hbr | Physical-Virtual | -2.316 | 2.05 | -1.13 | 84 | 0.6419 | 5.146 | 0.62 |
| 1 | 1 | hbr | Physical-MR | -4.53 | 2.105 | -2.152 | 84 | 0.1946 | 5.282 | 0.604 |
| 1 | 1 | hbr | MR-Virtual | 2.214 | 1.946 | 1.138 | 84 | 0.6419 | 4.884 | 0.654 |
| 1 | 2 | hbo | Physical-Virtual | -2.774 | 2.229 | -1.245 | 84 | 0.5739 | 5.593 | 0.571 |
| 1 | 2 | hbo | Physical-MR | -0.39 | 2.266 | -0.172 | 84 | 0.9328 | 5.688 | 0.561 |
| 1 | 2 | hbo | MR-Virtual | -2.383 | 2.272 | -1.049 | 84 | 0.6685 | 5.701 | 0.56 |
| 1 | 2 | hbr | Physical-Virtual | -0.602 | 1.652 | -0.364 | 84 | 0.8411 | 4.146 | 0.77 |
| 1 | 2 | hbr | Physical-MR | 0.163 | 1.611 | 0.101 | 84 | 0.9539 | 4.044 | 0.789 |
| 1 | 2 | hbr | MR-Virtual | -0.765 | 1.638 | -0.467 | 84 | 0.8299 | 4.11 | 0.777 |
| 2 | 1 | hbo | Physical-Virtual | -9.917 | 2.139 | -4.638 | 84 | 0.0014* | 5.367 | 0.595 |
| 2 | 1 | hbo | Physical-MR | -9.508 | 2.231 | -4.262 | 84 | 0.0028* | 5.599 | 0.57 |
| 2 | 1 | hbo | MR-Virtual | -0.409 | 2.049 | -0.2 | 84 | 0.9188 | 5.143 | 0.621 |
| 2 | 1 | hbr | Physical-Virtual | -3.873 | 1.272 | -3.045 | 84 | 0.0672 | 3.192 | 1 |
| 2 | 1 | hbr | Physical-MR | -0.505 | 1.334 | -0.379 | 84 | 0.8411 | 3.347 | 0.954 |
| 2 | 1 | hbr | MR-Virtual | -3.368 | 1.285 | -2.622 | 84 | 0.1019 | 3.224 | 0.99 |
| 2 | 2 | hbo | Physical-Virtual | -5.326 | 2.011 | -2.648 | 84 | 0.1019 | 5.047 | 0.633 |
| 2 | 2 | hbo | Physical-MR | -4.422 | 1.971 | -2.244 | 84 | 0.1946 | 4.946 | 0.645 |
| 2 | 2 | hbo | MR-Virtual | -0.904 | 1.961 | -0.461 | 84 | 0.8299 | 4.921 | 0.649 |
| 2 | 2 | hbr | Physical-Virtual | 0.416 | 1.383 | 0.301 | 84 | 0.8599 | 3.471 | 0.92 |
| 2 | 2 | hbr | Physical-MR | 1.425 | 1.324 | 1.077 | 84 | 0.654 | 3.322 | 0.961 |
| 2 | 2 | hbr | MR-Virtual | -1.009 | 1.342 | -0.752 | 84 | 0.7782 | 3.367 | 0.948 |
| 2 | 3 | hbo | Physical-Virtual | -5.374 | 2.704 | -1.987 | 84 | 0.2355 | 6.786 | 0.47 |
| 2 | 3 | hbo | Physical-MR | -7.236 | 2.616 | -2.767 | 84 | 0.1019 | 6.564 | 0.486 |
| 2 | 3 | hbo | MR-Virtual | 1.862 | 2.438 | 0.764 | 84 | 0.7782 | 6.119 | 0.522 |
| 2 | 3 | hbr | Physical-Virtual | 3.239 | 1.858 | 1.743 | 84 | 0.3481 | 4.662 | 0.685 |
| 2 | 3 | hbr | Physical-MR | 4.119 | 1.842 | 2.236 | 84 | 0.1946 | 4.623 | 0.69 |
| 2 | 3 | hbr | MR-Virtual | -0.88 | 1.669 | -0.527 | 84 | 0.8299 | 4.189 | 0.762 |
| 3 | 1 | hbo | Physical-Virtual | 2.288 | 2.572 | 0.89 | 84 | 0.7591 | 6.454 | 0.495 |
| 3 | 1 | hbo | Physical-MR | -1.398 | 2.433 | -0.575 | 84 | 0.8299 | 6.105 | 0.523 |
| 3 | 1 | hbo | MR-Virtual | 3.685 | 2.467 | 1.494 | 84 | 0.4692 | 6.192 | 0.516 |
| 3 | 1 | hbr | Physical-Virtual | 4.503 | 1.648 | 2.732 | 84 | 0.1019 | 4.136 | 0.772 |
| 3 | 1 | hbr | Physical-MR | 2.549 | 1.647 | 1.548 | 84 | 0.4515 | 4.132 | 0.773 |
| 3 | 1 | hbr | MR-Virtual | 1.954 | 1.573 | 1.243 | 84 | 0.5739 | 3.947 | 0.809 |
| 3 | 3 | hbo | Physical-Virtual | -5.106 | 2.761 | -1.849 | 84 | 0.3057 | 6.93 | 0.461 |
| 3 | 3 | hbo | Physical-MR | -1.1 | 2.505 | -0.439 | 84 | 0.8309 | 6.288 | 0.508 |
| 3 | 3 | hbo | MR-Virtual | -4.006 | 2.442 | -1.64 | 84 | 0.4038 | 6.129 | 0.521 |
| 3 | 3 | hbr | Physical-Virtual | 1.525 | 1.894 | 0.805 | 84 | 0.7782 | 4.754 | 0.671 |
| 3 | 3 | hbr | Physical-MR | 3.258 | 1.82 | 1.79 | 84 | 0.3326 | 4.567 | 0.699 |
| 3 | 3 | hbr | MR-Virtual | -1.734 | 1.73 | -1.002 | 84 | 0.7035 | 4.341 | 0.735 |
| 4 | 2 | hbo | Physical-Virtual | -5.515 | 2.489 | -2.216 | 84 | 0.1946 | 6.247 | 0.511 |
| 4 | 2 | hbo | Physical-MR | -6.473 | 2.468 | -2.623 | 84 | 0.1019 | 6.194 | 0.515 |
| 4 | 2 | hbo | MR-Virtual | 0.958 | 2.116 | 0.452 | 84 | 0.8299 | 5.312 | 0.601 |
| 4 | 2 | hbr | Physical-Virtual | 2.833 | 1.802 | 1.572 | 84 | 0.4456 | 4.523 | 0.706 |
| 4 | 2 | hbr | Physical-MR | 1.529 | 1.756 | 0.87 | 84 | 0.7591 | 4.408 | 0.724 |
| 4 | 2 | hbr | MR-Virtual | 1.305 | 1.691 | 0.771 | 84 | 0.7782 | 4.245 | 0.752 |
| 4 | 3 | hbo | Physical-Virtual | -5.795 | 2.742 | -2.114 | 84 | 0.2026 | 6.881 | 0.464 |
| 4 | 3 | hbo | Physical-MR | -8.907 | 2.573 | -3.461 | 84 | 0.0229* | 6.458 | 0.494 |
| 4 | 3 | hbo | MR-Virtual | 3.112 | 2.47 | 1.26 | 84 | 0.5739 | 6.199 | 0.515 |
| 4 | 3 | hbr | Physical-Virtual | 0.269 | 1.977 | 0.136 | 84 | 0.9504 | 4.962 | 0.643 |
| 4 | 3 | hbr | Physical-MR | 1.176 | 1.96 | 0.6 | 84 | 0.8299 | 4.918 | 0.649 |
| 4 | 3 | hbr | MR-Virtual | -0.907 | 1.851 | -0.49 | 84 | 0.8299 | 4.646 | 0.687 |
| 5 | 4 | hbo | Physical-Virtual | 4.435 | 5.504 | 0.806 | 84 | 0.7782 | 13.81 | 0.231 |
| 5 | 4 | hbo | Physical-MR | -5.773 | 5.172 | -1.116 | 84 | 0.6419 | 12.98 | 0.246 |
| 5 | 4 | hbo | MR-Virtual | 10.21 | 5.041 | 2.025 | 84 | 0.2326 | 12.65 | 0.252 |
| 5 | 4 | hbr | Physical-Virtual | -3.942 | 3.083 | -1.279 | 84 | 0.5739 | 7.736 | 0.413 |
| 5 | 4 | hbr | Physical-MR | 1.117 | 2.87 | 0.389 | 84 | 0.8411 | 7.202 | 0.443 |
| 5 | 4 | hbr | MR-Virtual | -5.059 | 2.922 | -1.732 | 84 | 0.3481 | 7.332 | 0.435 |
| 5 | 5 | hbo | Physical-Virtual | -4.855 | 2.235 | -2.173 | 84 | 0.1946 | 5.608 | 0.569 |
| 5 | 5 | hbo | Physical-MR | -2.054 | 2.329 | -0.882 | 84 | 0.7591 | 5.846 | 0.546 |
| 5 | 5 | hbo | MR-Virtual | -2.801 | 2.184 | -1.283 | 84 | 0.5739 | 5.481 | 0.582 |
| 5 | 5 | hbr | Physical-Virtual | 0.5 | 1.58 | 0.316 | 84 | 0.8556 | 3.965 | 0.805 |
| 5 | 5 | hbr | Physical-MR | -0.219 | 1.701 | -0.129 | 84 | 0.9504 | 4.268 | 0.748 |
| 5 | 5 | hbr | MR-Virtual | 0.718 | 1.593 | 0.451 | 84 | 0.8299 | 3.998 | 0.798 |
| 6 | 4 | hbo | Physical-Virtual | -1.649 | 2.933 | -0.562 | 84 | 0.8299 | 7.362 | 0.434 |
| 6 | 4 | hbo | Physical-MR | -2.56 | 2.6 | -0.984 | 84 | 0.7038 | 6.525 | 0.489 |
| 6 | 4 | hbo | MR-Virtual | 0.911 | 2.872 | 0.317 | 84 | 0.8556 | 7.209 | 0.443 |
| 6 | 4 | hbr | Physical-Virtual | 3.357 | 1.548 | 2.169 | 84 | 0.1946 | 3.884 | 0.822 |
| 6 | 4 | hbr | Physical-MR | -1.066 | 1.722 | -0.619 | 84 | 0.8299 | 4.321 | 0.739 |
| 6 | 4 | hbr | MR-Virtual | 4.423 | 1.642 | 2.693 | 84 | 0.1019 | 4.121 | 0.775 |
| 6 | 5 | hbo | Physical-Virtual | -0.785 | 2.132 | -0.368 | 84 | 0.8411 | 5.352 | 0.597 |
| 6 | 5 | hbo | Physical-MR | -0.986 | 2.146 | -0.459 | 84 | 0.8299 | 5.386 | 0.593 |
| 6 | 5 | hbo | MR-Virtual | 0.201 | 2.195 | 0.091 | 84 | 0.9539 | 5.51 | 0.579 |
| 6 | 5 | hbr | Physical-Virtual | -1.868 | 1.329 | -1.406 | 84 | 0.5046 | 3.336 | 0.957 |
| 6 | 5 | hbr | Physical-MR | -1.36 | 1.394 | -0.975 | 84 | 0.7038 | 3.5 | 0.912 |
| 6 | 5 | hbr | MR-Virtual | -0.509 | 1.379 | -0.369 | 84 | 0.8411 | 3.46 | 0.923 |
| 6 | 6 | hbo | Physical-Virtual | -1.964 | 3.601 | -0.545 | 84 | 0.8299 | 9.038 | 0.353 |
| 6 | 6 | hbo | Physical-MR | -2.774 | 3.548 | -0.782 | 84 | 0.7782 | 8.904 | 0.359 |
| 6 | 6 | hbo | MR-Virtual | 0.81 | 3.523 | 0.23 | 84 | 0.9022 | 8.842 | 0.361 |
| 6 | 6 | hbr | Physical-Virtual | 1.518 | 2.258 | 0.672 | 84 | 0.8299 | 5.667 | 0.563 |
| 6 | 6 | hbr | Physical-MR | 0.644 | 2.325 | 0.277 | 84 | 0.8711 | 5.836 | 0.547 |
| 6 | 6 | hbr | MR-Virtual | 0.874 | 2.351 | 0.372 | 84 | 0.8411 | 5.9 | 0.541 |
| 7 | 4 | hbo | Physical-Virtual | 11.9 | 3.151 | 3.777 | 84 | 0.0106* | 7.908 | 0.404 |
| 7 | 4 | hbo | Physical-MR | 7.067 | 3.12 | 2.265 | 84 | 0.1946 | 7.831 | 0.408 |
| 7 | 4 | hbo | MR-Virtual | 4.834 | 3.213 | 1.505 | 84 | 0.4692 | 8.063 | 0.396 |
| 7 | 4 | hbr | Physical-Virtual | 2.794 | 1.894 | 1.475 | 84 | 0.4709 | 4.754 | 0.672 |
| 7 | 4 | hbr | Physical-MR | -1.67 | 2.087 | -0.8 | 84 | 0.7782 | 5.238 | 0.609 |
| 7 | 4 | hbr | MR-Virtual | 4.464 | 1.826 | 2.444 | 84 | 0.1494 | 4.583 | 0.697 |
| 7 | 6 | hbo | Physical-Virtual | -0.298 | 2.523 | -0.118 | 84 | 0.9504 | 6.333 | 0.504 |
| 7 | 6 | hbo | Physical-MR | -2.047 | 2.629 | -0.779 | 84 | 0.7782 | 6.597 | 0.484 |
| 7 | 6 | hbo | MR-Virtual | 1.749 | 2.693 | 0.649 | 84 | 0.8299 | 6.758 | 0.472 |
| 7 | 6 | hbr | Physical-Virtual | 1.654 | 1.756 | 0.942 | 84 | 0.7248 | 4.407 | 0.724 |
| 7 | 6 | hbr | Physical-MR | 0.619 | 1.859 | 0.333 | 84 | 0.8556 | 4.666 | 0.684 |
| 7 | 6 | hbr | MR-Virtual | 1.035 | 1.877 | 0.551 | 84 | 0.8299 | 4.711 | 0.678 |
| 8 | 5 | hbo | Physical-Virtual | -3.702 | 3.209 | -1.153 | 84 | 0.6419 | 8.055 | 0.396 |
| 8 | 5 | hbo | Physical-MR | -3.881 | 3.126 | -1.242 | 84 | 0.5739 | 7.846 | 0.407 |
| 8 | 5 | hbo | MR-Virtual | 0.18 | 2.752 | 0.065 | 84 | 0.958 | 6.908 | 0.462 |
| 8 | 5 | hbr | Physical-Virtual | 2.235 | 2.044 | 1.094 | 84 | 0.651 | 5.129 | 0.622 |
| 8 | 5 | hbr | Physical-MR | 1.264 | 2.002 | 0.631 | 84 | 0.8299 | 5.023 | 0.636 |
| 8 | 5 | hbr | MR-Virtual | 0.971 | 1.973 | 0.492 | 84 | 0.8299 | 4.952 | 0.645 |
| 8 | 6 | hbo | Physical-Virtual | -5.073 | 3.529 | -1.437 | 84 | 0.4901 | 8.858 | 0.36 |
| 8 | 6 | hbo | Physical-MR | -6.786 | 3.372 | -2.012 | 84 | 0.2326 | 8.463 | 0.377 |
| 8 | 6 | hbo | MR-Virtual | 1.712 | 3.273 | 0.523 | 84 | 0.8299 | 8.215 | 0.389 |
| 8 | 6 | hbr | Physical-Virtual | 1.425 | 2.287 | 0.623 | 84 | 0.8299 | 5.739 | 0.556 |
| 8 | 6 | hbr | Physical-MR | 0.044 | 2.266 | 0.02 | 84 | 0.9844 | 5.686 | 0.561 |
| 8 | 6 | hbr | MR-Virtual | 1.38 | 2.237 | 0.617 | 84 | 0.8299 | 5.613 | 0.569 |

## Glm3: Between-group differences in activation during design change task

Table 5 Contrast statistics for between group-differences in activation during design change task. Significant channels (q<0.05) are highlighted in green with an asterisk (*).

| source | detector | type | contrast | beta | se | t-stat | dfe | q | Min  Discoverable  Change | Relative  Power |
| --- | --- | --- | --- | --- | --- | --- | --- | --- | --- | --- |
| 1 | 1 | hbo | Physical-Virtual | -3.856 | 3.746 | -1.0294 | 85 | 0.5126 | 9.399 | 0.354 |
| 1 | 1 | hbo | Physical-MR | 5.263 | 3.269 | 1.6101 | 85 | 0.3856 | 8.203 | 0.405 |
| 1 | 1 | hbo | MR-Virtual | -9.119 | 3.35 | -2.722 | 85 | 0.0922 | 8.407 | 0.395 |
| 1 | 1 | hbr | Physical-Virtual | 2.337 | 2.267 | 1.0312 | 85 | 0.5126 | 5.688 | 0.584 |
| 1 | 1 | hbr | Physical-MR | -0.519 | 2.107 | -0.2465 | 85 | 0.9119 | 5.287 | 0.629 |
| 1 | 1 | hbr | MR-Virtual | 2.857 | 2.222 | 1.2856 | 85 | 0.4744 | 5.576 | 0.596 |
| 1 | 2 | hbo | Physical-Virtual | -0.524 | 2.599 | -0.2018 | 85 | 0.917 | 6.521 | 0.51 |
| 1 | 2 | hbo | Physical-MR | 1.138 | 2.47 | 0.4609 | 85 | 0.8208 | 6.198 | 0.536 |
| 1 | 2 | hbo | MR-Virtual | -1.663 | 2.37 | -0.7016 | 85 | 0.6818 | 5.948 | 0.559 |
| 1 | 2 | hbr | Physical-Virtual | -0.753 | 1.721 | -0.4373 | 85 | 0.8326 | 4.319 | 0.77 |
| 1 | 2 | hbr | Physical-MR | -3.603 | 1.625 | -2.2165 | 85 | 0.1913 | 4.079 | 0.815 |
| 1 | 2 | hbr | MR-Virtual | 2.85 | 1.619 | 1.7604 | 85 | 0.3277 | 4.063 | 0.818 |
| 2 | 1 | hbo | Physical-Virtual | -2.847 | 2.482 | -1.147 | 85 | 0.5092 | 6.229 | 0.534 |
| 2 | 1 | hbo | Physical-MR | -0.084 | 2.434 | -0.0345 | 85 | 0.9803 | 6.109 | 0.544 |
| 2 | 1 | hbo | MR-Virtual | -2.763 | 2.368 | -1.1666 | 85 | 0.5026 | 5.943 | 0.559 |
| 2 | 1 | hbr | Physical-Virtual | -0.155 | 1.388 | -0.1114 | 85 | 0.9391 | 3.484 | 0.954 |
| 2 | 1 | hbr | Physical-MR | -1.153 | 1.411 | -0.8171 | 85 | 0.633 | 3.54 | 0.939 |
| 2 | 1 | hbr | MR-Virtual | 0.998 | 1.368 | 0.7297 | 85 | 0.6818 | 3.433 | 0.968 |
| 2 | 2 | hbo | Physical-Virtual | 2.175 | 2.144 | 1.0145 | 85 | 0.5126 | 5.38 | 0.618 |
| 2 | 2 | hbo | Physical-MR | 1.851 | 2.161 | 0.8565 | 85 | 0.608 | 5.424 | 0.613 |
| 2 | 2 | hbo | MR-Virtual | 0.324 | 2.201 | 0.1471 | 85 | 0.9263 | 5.524 | 0.602 |
| 2 | 2 | hbr | Physical-Virtual | 0.462 | 1.407 | 0.3281 | 85 | 0.8888 | 3.529 | 0.942 |
| 2 | 2 | hbr | Physical-MR | -1.922 | 1.325 | -1.4508 | 85 | 0.4076 | 3.324 | 1 |
| 2 | 2 | hbr | MR-Virtual | 2.383 | 1.392 | 1.7117 | 85 | 0.3379 | 3.494 | 0.951 |
| 2 | 3 | hbo | Physical-Virtual | 4.984 | 3.177 | 1.5688 | 85 | 0.3856 | 7.971 | 0.417 |
| 2 | 3 | hbo | Physical-MR | 6.227 | 3.126 | 1.9924 | 85 | 0.2432 | 7.843 | 0.424 |
| 2 | 3 | hbo | MR-Virtual | -1.244 | 2.954 | -0.4211 | 85 | 0.8376 | 7.412 | 0.448 |
| 2 | 3 | hbr | Physical-Virtual | -0.982 | 1.834 | -0.5355 | 85 | 0.7725 | 4.602 | 0.722 |
| 2 | 3 | hbr | Physical-MR | -3.287 | 1.797 | -1.8292 | 85 | 0.3029 | 4.509 | 0.737 |
| 2 | 3 | hbr | MR-Virtual | 2.305 | 1.755 | 1.3135 | 85 | 0.4621 | 4.403 | 0.755 |
| 3 | 1 | hbo | Physical-Virtual | 8.627 | 2.873 | 3.0027 | 85 | 0.0865 | 7.209 | 0.461 |
| 3 | 1 | hbo | Physical-MR | 8.691 | 2.938 | 2.9583 | 85 | 0.0865 | 7.372 | 0.451 |
| 3 | 1 | hbo | MR-Virtual | -0.064 | 2.603 | -0.0247 | 85 | 0.9803 | 6.532 | 0.509 |
| 3 | 1 | hbr | Physical-Virtual | 2.028 | 1.643 | 1.2348 | 85 | 0.4856 | 4.122 | 0.806 |
| 3 | 1 | hbr | Physical-MR | 4.632 | 1.72 | 2.6927 | 85 | 0.0922 | 4.317 | 0.77 |
| 3 | 1 | hbr | MR-Virtual | -2.604 | 1.619 | -1.6086 | 85 | 0.3856 | 4.062 | 0.818 |
| 3 | 3 | hbo | Physical-Virtual | 1.876 | 2.921 | 0.6422 | 85 | 0.7143 | 7.329 | 0.454 |
| 3 | 3 | hbo | Physical-MR | 3.221 | 2.88 | 1.1185 | 85 | 0.5126 | 7.226 | 0.46 |
| 3 | 3 | hbo | MR-Virtual | -1.345 | 2.789 | -0.4822 | 85 | 0.8111 | 6.999 | 0.475 |
| 3 | 3 | hbr | Physical-Virtual | -0.213 | 1.948 | -0.1095 | 85 | 0.9391 | 4.888 | 0.68 |
| 3 | 3 | hbr | Physical-MR | 0.46 | 1.912 | 0.2405 | 85 | 0.9119 | 4.798 | 0.693 |
| 3 | 3 | hbr | MR-Virtual | -0.673 | 1.854 | -0.3631 | 85 | 0.8706 | 4.652 | 0.715 |
| 4 | 2 | hbo | Physical-Virtual | 3.224 | 2.664 | 1.2102 | 85 | 0.4861 | 6.684 | 0.497 |
| 4 | 2 | hbo | Physical-MR | 5.606 | 2.731 | 2.0528 | 85 | 0.222 | 6.852 | 0.485 |
| 4 | 2 | hbo | MR-Virtual | -2.382 | 2.308 | -1.0319 | 85 | 0.5126 | 5.792 | 0.574 |
| 4 | 2 | hbr | Physical-Virtual | -2.742 | 1.853 | -1.4799 | 85 | 0.4052 | 4.649 | 0.715 |
| 4 | 2 | hbr | Physical-MR | -2.476 | 1.74 | -1.4225 | 85 | 0.4176 | 4.367 | 0.761 |
| 4 | 2 | hbr | MR-Virtual | -0.266 | 1.64 | -0.1622 | 85 | 0.9242 | 4.117 | 0.807 |
| 4 | 3 | hbo | Physical-Virtual | 4.272 | 3.187 | 1.3402 | 85 | 0.4549 | 7.999 | 0.416 |
| 4 | 3 | hbo | Physical-MR | 8.118 | 2.86 | 2.8382 | 85 | 0.0875 | 7.177 | 0.463 |
| 4 | 3 | hbo | MR-Virtual | -3.846 | 2.852 | -1.3483 | 85 | 0.4549 | 7.157 | 0.464 |
| 4 | 3 | hbr | Physical-Virtual | 1.266 | 1.942 | 0.6518 | 85 | 0.7143 | 4.873 | 0.682 |
| 4 | 3 | hbr | Physical-MR | -3.959 | 1.815 | -2.1819 | 85 | 0.1913 | 4.554 | 0.73 |
| 4 | 3 | hbr | MR-Virtual | 5.225 | 1.818 | 2.8743 | 85 | 0.0875 | 4.562 | 0.729 |
| 5 | 4 | hbo | Physical-Virtual | 12.43 | 5.406 | 2.2993 | 85 | 0.1847 | 13.57 | 0.245 |
| 5 | 4 | hbo | Physical-MR | 7.204 | 4.562 | 1.5791 | 85 | 0.3856 | 11.45 | 0.29 |
| 5 | 4 | hbo | MR-Virtual | 5.227 | 4.984 | 1.0488 | 85 | 0.5126 | 12.51 | 0.266 |
| 5 | 4 | hbr | Physical-Virtual | 1.068 | 3.424 | 0.312 | 85 | 0.8888 | 8.593 | 0.387 |
| 5 | 4 | hbr | Physical-MR | -2.21 | 2.972 | -0.7437 | 85 | 0.6793 | 7.459 | 0.446 |
| 5 | 4 | hbr | MR-Virtual | 3.279 | 3.247 | 1.0098 | 85 | 0.5126 | 8.147 | 0.408 |
| 5 | 5 | hbo | Physical-Virtual | -0.067 | 2.35 | -0.0285 | 85 | 0.9803 | 5.898 | 0.564 |
| 5 | 5 | hbo | Physical-MR | 5.765 | 2.332 | 2.4721 | 85 | 0.1433 | 5.852 | 0.568 |
| 5 | 5 | hbo | MR-Virtual | -5.832 | 2.379 | -2.4512 | 85 | 0.1433 | 5.97 | 0.557 |
| 5 | 5 | hbr | Physical-Virtual | -0.291 | 1.673 | -0.1741 | 85 | 0.9242 | 4.197 | 0.792 |
| 5 | 5 | hbr | Physical-MR | -3.823 | 1.738 | -2.1993 | 85 | 0.1913 | 4.362 | 0.762 |
| 5 | 5 | hbr | MR-Virtual | 3.531 | 1.717 | 2.0565 | 85 | 0.222 | 4.309 | 0.771 |
| 6 | 4 | hbo | Physical-Virtual | 1.688 | 2.928 | 0.5764 | 85 | 0.7545 | 7.347 | 0.452 |
| 6 | 4 | hbo | Physical-MR | -3.154 | 2.864 | -1.1013 | 85 | 0.5126 | 7.186 | 0.463 |
| 6 | 4 | hbo | MR-Virtual | 4.841 | 2.663 | 1.8179 | 85 | 0.3029 | 6.683 | 0.497 |
| 6 | 4 | hbr | Physical-Virtual | 2.517 | 1.737 | 1.4492 | 85 | 0.4076 | 4.358 | 0.763 |
| 6 | 4 | hbr | Physical-MR | 0.27 | 1.682 | 0.1605 | 85 | 0.9242 | 4.221 | 0.788 |
| 6 | 4 | hbr | MR-Virtual | 2.247 | 1.798 | 1.2496 | 85 | 0.4856 | 4.511 | 0.737 |
| 6 | 5 | hbo | Physical-Virtual | 2.728 | 2.323 | 1.1742 | 85 | 0.5026 | 5.83 | 0.57 |
| 6 | 5 | hbo | Physical-MR | 2.174 | 2.47 | 0.8799 | 85 | 0.597 | 6.199 | 0.536 |
| 6 | 5 | hbo | MR-Virtual | 0.555 | 2.622 | 0.2115 | 85 | 0.917 | 6.579 | 0.505 |
| 6 | 5 | hbr | Physical-Virtual | 1.814 | 1.358 | 1.3354 | 85 | 0.4549 | 3.408 | 0.975 |
| 6 | 5 | hbr | Physical-MR | -1.523 | 1.447 | -1.0522 | 85 | 0.5126 | 3.632 | 0.915 |
| 6 | 5 | hbr | MR-Virtual | 3.337 | 1.473 | 2.2658 | 85 | 0.1872 | 3.695 | 0.9 |
| 6 | 6 | hbo | Physical-Virtual | 4.89 | 3.978 | 1.2294 | 85 | 0.4856 | 9.982 | 0.333 |
| 6 | 6 | hbo | Physical-MR | -2.741 | 3.918 | -0.6996 | 85 | 0.6818 | 9.833 | 0.338 |
| 6 | 6 | hbo | MR-Virtual | 7.632 | 3.716 | 2.0535 | 85 | 0.222 | 9.326 | 0.356 |
| 6 | 6 | hbr | Physical-Virtual | -3.665 | 2.474 | -1.4814 | 85 | 0.4052 | 6.208 | 0.535 |
| 6 | 6 | hbr | Physical-MR | -2.975 | 2.433 | -1.2227 | 85 | 0.4856 | 6.106 | 0.544 |
| 6 | 6 | hbr | MR-Virtual | -0.689 | 2.411 | -0.2859 | 85 | 0.9007 | 6.05 | 0.549 |
| 7 | 4 | hbo | Physical-Virtual | 0.687 | 3.211 | 0.2139 | 85 | 0.917 | 8.058 | 0.412 |
| 7 | 4 | hbo | Physical-MR | 12.66 | 3.286 | 3.8534 | 85 | 0.0122* | 8.247 | 0.403 |
| 7 | 4 | hbo | MR-Virtual | -11.98 | 2.943 | -4.0701 | 85 | 0.0113* | 7.384 | 0.45 |
| 7 | 4 | hbr | Physical-Virtual | -3.074 | 1.986 | -1.5476 | 85 | 0.3871 | 4.984 | 0.667 |
| 7 | 4 | hbr | Physical-MR | -1.911 | 2.013 | -0.9493 | 85 | 0.5482 | 5.051 | 0.658 |
| 7 | 4 | hbr | MR-Virtual | -1.163 | 1.859 | -0.6255 | 85 | 0.72 | 4.665 | 0.713 |
| 7 | 6 | hbo | Physical-Virtual | 5.635 | 2.978 | 1.8919 | 85 | 0.2907 | 7.474 | 0.445 |
| 7 | 6 | hbo | Physical-MR | 2.28 | 3.197 | 0.7133 | 85 | 0.6818 | 8.021 | 0.414 |
| 7 | 6 | hbo | MR-Virtual | 3.354 | 3.033 | 1.106 | 85 | 0.5126 | 7.611 | 0.437 |
| 7 | 6 | hbr | Physical-Virtual | -2.099 | 1.961 | -1.0702 | 85 | 0.5126 | 4.921 | 0.675 |
| 7 | 6 | hbr | Physical-MR | 3.493 | 2.041 | 1.711 | 85 | 0.3379 | 5.122 | 0.649 |
| 7 | 6 | hbr | MR-Virtual | -5.592 | 2.035 | -2.7475 | 85 | 0.0922 | 5.107 | 0.651 |
| 8 | 5 | hbo | Physical-Virtual | -4.719 | 3.016 | -1.5646 | 85 | 0.3856 | 7.568 | 0.439 |
| 8 | 5 | hbo | Physical-MR | -3.1 | 3.086 | -1.0045 | 85 | 0.5126 | 7.745 | 0.429 |
| 8 | 5 | hbo | MR-Virtual | -1.618 | 2.998 | -0.5399 | 85 | 0.7725 | 7.523 | 0.442 |
| 8 | 5 | hbr | Physical-Virtual | 0.802 | 2.108 | 0.3806 | 85 | 0.8645 | 5.289 | 0.629 |
| 8 | 5 | hbr | Physical-MR | -0.66 | 2.128 | -0.3103 | 85 | 0.8888 | 5.34 | 0.622 |
| 8 | 5 | hbr | MR-Virtual | 1.462 | 1.892 | 0.773 | 85 | 0.6625 | 4.748 | 0.7 |
| 8 | 6 | hbo | Physical-Virtual | -5.192 | 3.474 | -1.4944 | 85 | 0.4052 | 8.718 | 0.381 |
| 8 | 6 | hbo | Physical-MR | -6.139 | 3.381 | -1.8158 | 85 | 0.3029 | 8.483 | 0.392 |
| 8 | 6 | hbo | MR-Virtual | 0.947 | 3.663 | 0.2585 | 85 | 0.9119 | 9.192 | 0.362 |
| 8 | 6 | hbr | Physical-Virtual | 2.433 | 2.337 | 1.0411 | 85 | 0.5126 | 5.864 | 0.567 |
| 8 | 6 | hbr | Physical-MR | -5.361 | 2.207 | -2.4291 | 85 | 0.1433 | 5.538 | 0.6 |
| 8 | 6 | hbr | MR-Virtual | 7.793 | 2.311 | 3.3728 | 85 | 0.0404* | 5.798 | 0.573 |

# Statistical details for workload and affective state

Please note, as robust model includes a bootstrap, numeric results will vary slightly each time the model is ran. Therefore, for example for Physical Demand, there might be slight discrepancy between numbers in the manuscript and here.

## Mental demand

Table 6 Descriptive Statistics for Mental Demand

| group | N | mental_demand_mean | mental_demand_sd | 95% CI lower | 95% CI upper |
| --- | --- | --- | --- | --- | --- |
| Control | 30 | 3.03 | 1.40 | 2.5 | 3.6 |
| Virtual | 30 | 3.93 | 2.45 | 3.0 | 4.8 |
| MR | 28 | 3.50 | 2.06 | 2.7 | 4.3 |

Table 7 Robust model results for Mental Demand

| Contrast | Estimated difference in Trimmed Means 𝜓 | 95% CI lower | 95% CI upper |
| --- | --- | --- | --- |
| Virtual vs. Physical | -0.50 | -2.22 | 0.67 |
| Virtual vs. MR | -0.22 | -1.44 | 0.67 |
| Physical vs. MR | 0.28 | -1.22 | 2.06 |

Effect size for omnibus robust test = 0.192

## Physical demand

Table 8 Descriptive Statistics for Physical Demand

| group | N | physical_demand_mean | physical_demand_sd | 95% CI lower | 95% CI upper |
| --- | --- | --- | --- | --- | --- |
| Physical | 30 | 3.23 | 2.21 | 2.4 | 4.1 |
| Virtual | 30 | 1.43 | 0.77 | 1.1 | 1.7 |
| MR | 28 | 3.11 | 1.57 | 2.5 | 3.7 |

Table 9 Robust model results for Physical Demand

| Contrast | Estimated difference in Trimmed Means 𝜓 | 95% CI lower | 95% CI upper |
| --- | --- | --- | --- |
| Virtual vs. Physical | 1.56 | 0.67 | 2.67 |
| Virtual vs. MR | -0.22 | -1.17 | 1.11 |
| Physical vs. MR | -1.78 | -2.39 | -1.11 |

Effect size for omnibus robust test = 0.545

Because of the discrepancy in results between the linear and the robust models we inspected this data further, see table and figure of trimmed means below.

The linear model assumptions are not meet (non-normality of residuals), and therefore it is appropriate to use a robust model. WRS2’s trimmed means procedures might behave this way because of the outliers, the skew and the variability of the data and which data exhibits which variability. As per Table 10, we see that the differences between the mean and the trimmed mean are substantial. The standard deviation is much smaller in the Virtual group than in the Physical and MR group. Once the data has been trimmed the three groups are much closer together.

Table 10 Trimmed compared to untrimmed mean for Physical Demand

| group | mean | Trimmed mean 0.2 | sd |
| --- | --- | --- | --- |
| Physical | 3.23 | 2.72 | 2.21 |
| Virtual | 1.43 | 1.17 | 0.77 |
| MR | 3.101 | 2.94 | 1.57 |

Table 11 Physical demand data plot. Left) complete data set, error bars represent a normal 95%CI. Right) Trimmed data set, error bars represent a bootstrapped 95%CI, which is what the robust model is using.


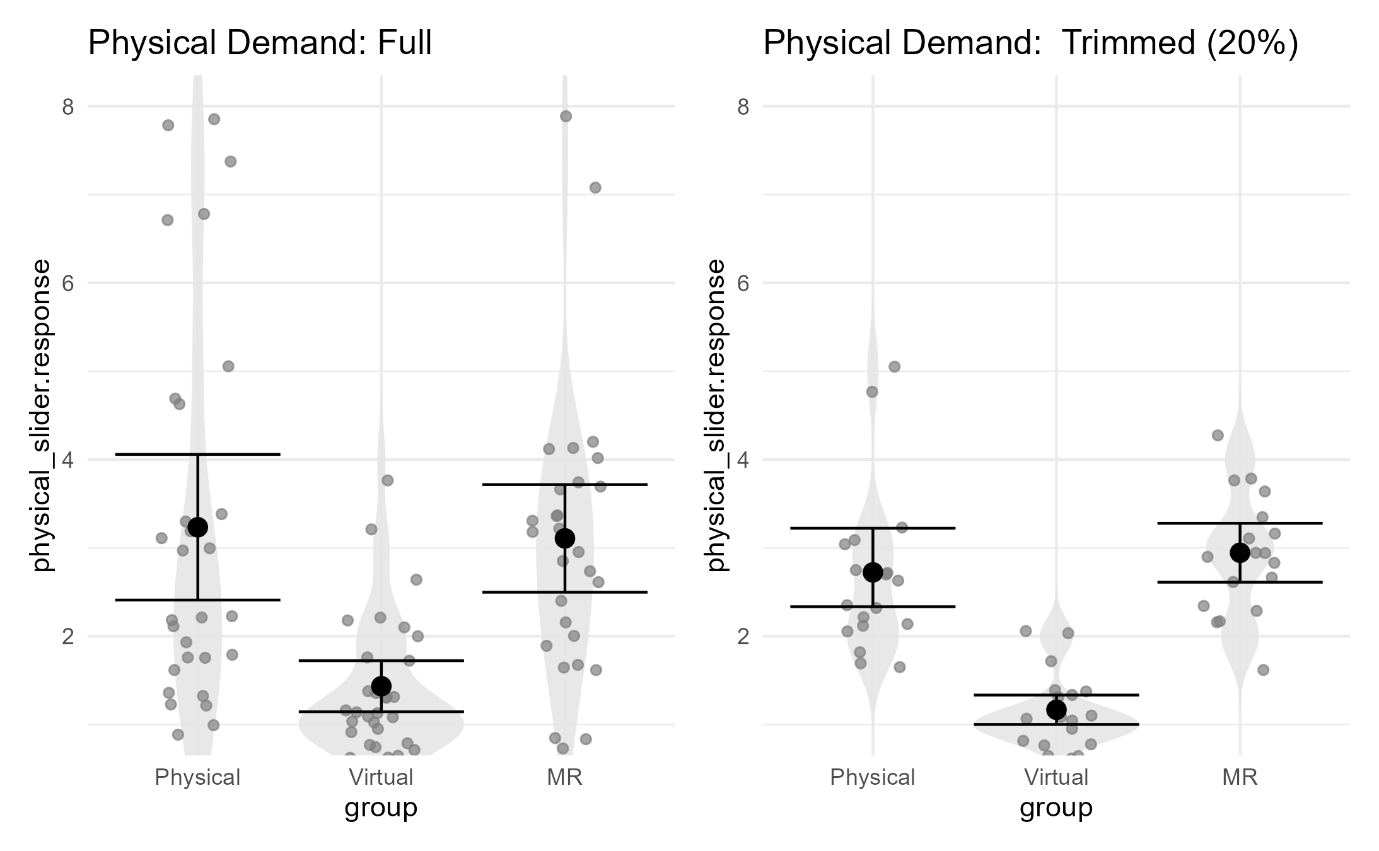


A third option is to use Kruskal-Wallis rank sum test, and post-hoc pair-wise comparisons with the Dunn test. We therefore ran the Kruskal–Wallis rank sum Test [stats::kruskal.test (R Core Team, 2024)] and post-hoc pairwise comparisons with the Dunn test [FSA::dunnTest (Ogle et al., 2025)] with a Holm correction (Holm, 1979). This test does not make assumptions about the distribution of the data and addresses if it is likely that an observation in one group is greater than an observation in some other group (Mangiafico, 2016). Epsilon squared [rcompanion::epsilonSquared (Mangiafico, 2025)] estimated effect size.

The results display significant differences between MR and Virtual, and Physical and Virtual, which corroborates what we see visually. We therefore rely on these results.

Table 12 Pairwise comparisons for Physical demand based on the Dunn test

| Comparison | Z | P.unadj | P.adj |
| --- | --- | --- | --- |
| MR - Physical | 0.6881484 | 4.913593e-01 | 4.913593e-01 |
| MR - Virtual | 4.7414945 | 2.121474e-06 | 6.364422e-06 |
| Physical - Virtual | 4.1250923 | 3.705860e-05 | 7.411721e-05 |

epsilon.squared = 0.307

## Temporal demand

Table 13 Descriptive Statistics for Temporal Demand

| group | N | temporal_demand_mean | temporal_demand_sd | 95% CI lower | 95% CI upper |
| --- | --- | --- | --- | --- | --- |
| Physical | 30 | 2.70 | 1.88 | 2.0 | 3.4 |
| Virtual | 30 | 3.03 | 2.34 | 2.2 | 3.9 |
| MR | 28 | 2.93 | 2.23 | 2.1 | 3.8 |

Table 14 Robust model results for Temporal Demand

| Contrast | Estimated difference in Trimmed Means 𝜓 | 95% CI lower | 95% CI upper |
| --- | --- | --- | --- |
| Virtual vs. Physical | -0.17 | -1.67 | 1.22 |
| Virtual vs. MR | -0.11 | -1.61 | 1.44 |
| Physical vs. MR | 0.06 | -1.56 | 1.61 |

Effect size for omnibus robust test = 0.061

## Performance

Table 15 Descriptive Statistics for Performance

| group | N | performance_mean | performance_sd | 95% CI lower | 95% CI upper |
| --- | --- | --- | --- | --- | --- |
| Physical | 30 | 3.73 | 2.18 | 2.9 | 4.5 |
| Virtual | 30 | 5.70 | 2.28 | 4.8 | 6.6 |
| MR | 28 | 4.71 | 2.27 | 3.8 | 5.6 |

Table 16 Linear model results for Performance

| Level1 | Level2 | Difference | 95% CI | SE | t(85) | p |
| --- | --- | --- | --- | --- | --- | --- |
| Virtual | MR | 0.99 | [-0.45, 2.43] | 0.59 | 1.67 | 0.295 |
| Physical | Virtual | -1.97 | [-3.38, -0.55] | 0.58 | -3.39 | 0.003 |
| Physical | MR | -0.98 | [-2.42, 0.46] | 0.59 | -1.66 | 0.299 |

Effect size, omega squared for omnibus F-test = 0.10

## Effort

Table 17 Descriptive Statistics for Effort

| group | N | effort_mean | effort_sd | 95% CI lower | 95% CI upper |
| --- | --- | --- | --- | --- | --- |
| Physical | 30 | 3.33 | 1.90 | 2.6 | 4.0 |
| Virtual | 30 | 3.23 | 1.83 | 2.5 | 3.9 |
| MR | 28 | 3.86 | 1.96 | 3.1 | 4.6 |

Table 18 Robust model results for Effort

| Contrast | Estimated difference in Trimmed Means 𝜓 | 95% CI lower | 95% CI upper |
| --- | --- | --- | --- |
| Virtual vs. Physical | 0.22 | -1.11 | 1.22 |
| Virtual vs. MR | -0.44 | -1.94 | 0.83 |
| Physical vs. MR | -0.67 | -1.83 | 0.50 |

Effect size for omnibus robust test = 0.185

## Frustration

Table 19 Descriptive Statistics for Frustration

| group | N | frustration_mean | frustration_sd | 95% CI lower | 95% CI upper |
| --- | --- | --- | --- | --- | --- |
| Physical | 30 | 2.80 | 2.20 | 2.0 | 3.6 |
| Virtual | 30 | 4.03 | 2.77 | 3.0 | 5.1 |
| MR | 28 | 2.46 | 1.90 | 1.7 | 3.2 |

Table 20 Robust model results for Frustration

| Contrast | Estimated difference in Trimmed Means 𝜓 | 95% CI lower | 95% CI upper |
| --- | --- | --- | --- |
| Virtual vs. Physical | -1.44 | -3.06 | 0.22 |
| Virtual vs. MR | 0.28 | -0.83 | 1.22 |
| Physical vs. MR | 1.72 | -0.06 | 3.39 |

Effect size for omnibus robust test = 0.396

## Overall Workload

Table 21 Descriptive Statistics for Overall Workload

| group | N | ow_mean | ow_sd | 95% CI lower | 95% CI upper |
| --- | --- | --- | --- | --- | --- |
| Physical | 30 | 2.60 | 1.54 | 2.0 | 3.2 |
| Virtual | 30 | 3.07 | 2.26 | 2.2 | 3.9 |
| MR | 28 | 3.46 | 2.10 | 2.7 | 4.3 |

Table 22 Robust model results for Overall Workload

| Contrast | Estimated difference in Trimmed Means 𝜓 | 95% CI lower | 95% CI upper |
| --- | --- | --- | --- |
| Virtual vs. Physical | -0.22 | -1.39 | 0.72 |
| Virtual vs. MR | -0.78 | -2.22 | 0.11 |
| Physical vs. MR | -0.56 | -2.11 | 0.78 |

Effect size for omnibus robust test = 0.298

## Stress

Table 23 Descriptive Statistics for Stress

| group | N | stress_mean | stress_sd | 95% CI lower | 95% CI upper |
| --- | --- | --- | --- | --- | --- |
| Physical | 30 | 2.53 | 1.80 | 1.9 | 3.2 |
| Virtual | 30 | 3.00 | 2.15 | 2.2 | 3.8 |
| MR | 28 | 2.39 | 1.79 | 1.7 | 3.1 |

Table 24 Robust model results for Stress

| Contrast | Estimated difference in Trimmed Means 𝜓 | 95% CI lower | 95% CI upper |
| --- | --- | --- | --- |
| Virtual vs. Physical | -0.39 | -1.50 | 0.78 |
| Virtual vs. MR | 0.17 | -0.94 | 1.44 |
| Physical vs. MR | 0.56 | -0.67 | 1.61 |

Effect size for omnibus robust test = 0.157

## Arousal

Table 25 Descriptive Statistics for Arousal

| group | N | arousal_mean | arousal_sd | 95% CI lower | 95% CI upper |
| --- | --- | --- | --- | --- | --- |
| Physical | 30 | 6.13 | 2.34 | 5.3 | 7.0 |
| Virtual | 30 | 6.80 | 1.86 | 6.1 | 7.5 |
| MR | 28 | 7.18 | 1.94 | 6.4 | 7.9 |

Table 26 Linear model results for Arousal

| Level1 | Level2 | Difference | 95% CI | SE | t(85) | p |
| --- | --- | --- | --- | --- | --- | --- |
| Virtual | MR | -0.38 | [-1.70, 0.95] | 0.54 | -0.70 | > .999 |
| Physical | Virtual | -0.67 | [-1.97, 0.64] | 0.53 | -1.25 | 0.644 |
| Physical | MR | -1.05 | [-2.37, 0.28] | 0.54 | -1.93 | 0.172 |

Effect size, omega squared for omnibus F-test = 0.02

## Valence

Table 27 Descriptive Statistics for Valence

| group | N | valence_mean | valence_sd | 95% CI lower | 95% CI upper |
| --- | --- | --- | --- | --- | --- |
| Physical | 30 | 8.27 | 1.66 | 7.6 | 8.9 |
| Virtual | 30 | 7.60 | 2.06 | 6.8 | 8.4 |
| MR | 28 | 8.21 | 1.32 | 7.7 | 8.7 |

Table 28 Linear model results for Valence

| Level1 | Level2 | Difference | 95% CI | SE | t(85) | p |
| --- | --- | --- | --- | --- | --- | --- |
| Virtual | MR | -0.61 | [-1.71, 0.49] | 0.45 | -1.36 | 0.529 |
| Physical | Virtual | 0.67 | [-0.41, 1.75] | 0.44 | 1.51 | 0.407 |
| Physical | MR | 0.05 | [-1.05, 1.15] | 0.45 | 0.12 | > .999 |

Effect size, omega squared for omnibus F-test = 8.80e-03

# Questionnaire - Subjective data collection:

Variables and questions

## Product questions

| **Variable** | **Question/Statement** | **Scale** |
| --- | --- | --- |
| mass_slider.response | The drill mass is appropriate for the specified task. | 1-7  (1, 2, 3, 4, 5, 6, 7)  ("Strongly disagree", "Strongly agree") |
| size_slider.response | The drill size is appropriate for the specified task. | 1-7  (1, 2, 3, 4, 5, 6, 7)  ("Strongly disagree", "Strongly agree") |
| task_well_slider.response | I expect the drill would perform the specified task well. | 1-7  (1, 2, 3, 4, 5, 6, 7)  ("Strongly disagree", "Strongly agree") |
| precision_slider.response | I expect I can use the drill with the appropriate precision. | 1-7  (1, 2, 3, 4, 5, 6, 7)  ("Strongly disagree", "Strongly agree") |
| interface_slider.response | The interface makes it easy to understand how to control the drill's operation. | 1-7  (1, 2, 3, 4, 5, 6, 7)  ("Strongly disagree", "Strongly agree") |
| battery_slider.response | It is easy to understand how to change the battery. | 1-7  (1, 2, 3, 4, 5, 6, 7)  ("Strongly disagree", "Strongly agree") |
| confidence_slider.response | How confident are you in your answers? | 1-7  (1, 2, 3, 4, 5, 6, 7)  ("Low confidence", "High confidence") |

## Technology questions

### System Usability Scale (SUS)

| **Variable** | **Question/Statement** | **Scale** |
| --- | --- | --- |
| sus1_slider.response | I think that I would like to use this technology frequently. | (1, 2, 3, 4, 5)  ("Strongly disagree", "Strongly agree") |
| sus2_slider.response | I found the technology unnecessarily complex. | (1, 2, 3, 4, 5)  ("Strongly disagree", "Strongly agree") |
| sus3_slider.response | I thought the technology was easy to use. | (1, 2, 3, 4, 5)  ("Strongly disagree", "Strongly agree") |
| sus4_slider.response | I think that I would need the support of a technical person to be able to use this technology. | (1, 2, 3, 4, 5)  ("Strongly disagree", "Strongly agree") |
| sus5_slider.response | I found the various functions in this technology were well integrated. | (1, 2, 3, 4, 5)  ("Strongly disagree", "Strongly agree") |
| sus6_slider.response | I thought there was too much inconsistency in this technology. | (1, 2, 3, 4, 5)  ("Strongly disagree", "Strongly agree") |
| sus7_slider.response | I would imagine that most people would learn to use this technology very quickly. | (1, 2, 3, 4, 5)  ("Strongly disagree", "Strongly agree") |
| sus8_slider.response | I found the technology very cumbersome to use. | (1, 2, 3, 4, 5)  ("Strongly disagree", "Strongly agree") |
| sus9_slider.response | I felt very confident using the technology. | (1, 2, 3, 4, 5)  ("Strongly disagree", "Strongly agree") |
| sus10_slider.response | I needed to learn a lot of things before I could get going with this technology. | (1, 2, 3, 4, 5)  ("Strongly disagree", "Strongly agree") |

### Knowledge dimensions and Affordances

| **Variable** | **Question/Statement** | **Scale** |
| --- | --- | --- |
| **Knowledge dimensions** | | |
| visualisation_slider.response | The representation the technology provides does well in informing my interpretations of the product. | (1, 2, 3, 4, 5, 6, 7)  ("Strongly disagree", "Strongly agree") |
| knowledge_management_slider.response | The technology does well in capturing how the prototype is used and why it is the way it is. | (1, 2, 3, 4, 5, 6, 7)  ("Strongly disagree", "Strongly agree") |
| **Affordances** | | |
| feedback_slider.response | When I use the prototype I quickly attain feedback that generates learning. | (1, 2, 3, 4, 5, 6, 7)  ("Strongly disagree", "Strongly agree") |
| flexibility_slider.response | The technology allows the prototype to change and supports exploration of other design options. | (1, 2, 3, 4, 5, 6, 7)  ("Strongly disagree", "Strongly agree") |
| fidelity_slider.response | The technology provides a realistic representation of the intended final product. | (1, 2, 3, 4, 5, 6, 7)  ("Strongly disagree", "Strongly agree") |
| analytic_capacity_slider.response | It was easy to perform active testing and analysis of the prototype. | (1, 2, 3, 4, 5, 6, 7)  ("Strongly disagree", "Strongly agree") |
| stakeholder_accessibility1_slider.response | This technology provided a broad opportunity for interaction. | (1, 2, 3, 4, 5, 6, 7)  ("Strongly disagree", "Strongly agree") |
| stakeholder_accessibility2_slider.response | This was a tangible and accessible experience. | (1, 2, 3, 4, 5, 6, 7)  ("Strongly disagree", "Strongly agree") |
| interactivity_slider.response | The technology allows a large range of interactions to be performed with the prototype. | (1, 2, 3, 4, 5, 6, 7)  ("Strongly disagree", "Strongly agree") |
| learning_slider.response | The technology enables broad learning regarding the prototype. | (1, 2, 3, 4, 5, 6, 7)  ("Strongly disagree", "Strongly agree") |

## Workload, affective state and stress

### NASA-TLX

| **Variable** | **Question/Statement** | **Scale** |
| --- | --- | --- |
| mental_slider.response | Mental Demand: How mentally demanding was the task? | (1, 2, 3, 4, 5, 6, 7, 8, 9, 10, 11)  "Very Low", "Very High" |
| physical_slider.response | Physical Demand: How physically demanding was the task? | (1, 2, 3, 4, 5, 6, 7, 8, 9, 10, 11)  "Very Low", "Very High" |
| temporal_slider.response | Temporal Demand: How hurried or rushed was the pace of the task? | (1, 2, 3, 4, 5, 6, 7, 8, 9, 10, 11)  "Very Low", "Very High" |
| performance_slider.response | Performance: How successful were you in accomplishing what you were asked to do? | (1, 2, 3, 4, 5, 6, 7, 8, 9, 10, 11)  "Perfect", "Failure" |
| effort_slider.response | Effort: How hard did you have to work to accomplish your level of performance? | (1, 2, 3, 4, 5, 6, 7, 8, 9, 10, 11)  "Very Low", "Very High" |
| frustration_slider.response | Frustration: How insecure, discouraged, irritated, stressed, and annoyed were you? | (1, 2, 3, 4, 5, 6, 7, 8, 9, 10, 11)  "Very Low", "Very High" |

### Affective state and stress

| **Variable** | **Question/Statement** | **Scale** |
| --- | --- | --- |
| ow_slider.response | Overall workload: How would you rate your overall workload? | (1, 2, 3, 4, 5, 6, 7, 8, 9, 10, 11)  "Very Low", "Very High" |
| stress_slider.response | Stress level: How stressed were you? | (1, 2, 3, 4, 5, 6, 7, 8, 9, 10, 11)  "Very Low", "Very High" |
| arousal_slider.response | Arousal: How alert, aroused, wide awake, or activated did you feel? | (1, 2, 3, 4, 5, 6, 7, 8, 9, 10, 11)  "Very Low (Sleepiness)", "Very High (Extreme alertness)" |
| valence_slider.response | Valence: How did you feel? | (1, 2, 3, 4, 5, 6, 7, 8, 9, 10, 11)  "Unpleasant feelings", "Pleasant feelings" |

## Demographics

| **Variable** | **Question/Statement** | **Scale** |
| --- | --- | --- |
| ageResp.text | What is your age?  Type your age here using whole numbers | years |
| sexResp.response | What is your biological sex? | (1,2,3)  ("Female", "Male", "Prefer not to say") |
| handednessResponse.response | What is your dominant hand? | (1,2,3)  ("Right", "Left", "Ambidextrous") |
| studentResp.response | Are you a student? | (1,2)  ("Yes", "No") |
| designExperienceResp.text | How many years of design/engineering experience do you have? Include completed years of related university study (e.g., engineering, design, or architecture) plus related industrial experience within this total. | years |
| coffeeResp.text | How many cups of coffee (or highly caffeinated drink) have you had today?  Type number of cups here using whole numbers | Number of cups |
| VR_experience_slider.response | What is your level of experience with Virtual Reality (VR) and VR headsets? | (1, 2, 3, 4, 5, 6, 7, 8, 9, 10, 11)  ("None - Never used VR before", "High - Extensive experience, use VR often") |
| CAD_experience_slider.response | What is your level of experience with Computer Aided Design (CAD) software? | (1, 2, 3, 4, 5, 6, 7, 8, 9, 10, 11)  ("None - Never used CAD before", "High - Extensive experience, use CAD often") |
| physical_prototyping_experience_slider .response | What is your level of experience with physical prototyping? | (1, 2, 3, 4, 5, 6, 7, 8, 9, 10, 11)  ("None - Never prototyped physically", "High - Extensive experience with physical prototyping") |
| drill_experience_slider.response | What is your level of experience with using a real power drill? | (1, 2, 3, 4, 5, 6, 7, 8, 9, 10, 11)  ("None - Never used a drill before", "High - Extensive experience, use them often") |

# References

Holm, S. (1979). A Simple Sequentially Rejective Multiple Test Procedure. *Scandinavian Journal of Statistics*, *6*(2), 65–70.

Mangiafico, S. S. (2016). *Summary and Analysis of Extension Program Evaluation in R, version 1.22.2, revised 2025*. rcompanion.org/handbook/.

Mangiafico, S. S. (2025). *rcompanion: Functions to Support Extension Education Program Evaluation*. Rutgers Cooperative Extension. https://CRAN.R-project.org/package=rcompanion/

Ogle, D. H., Doll, J. C., Wheeler, A. P., & Dinno, A. (2025). *FSA: Simple Fisheries Stock Assessment Methods* [Computer software]. https://CRAN.R-project.org/package=FSA

R Core Team. (2024). *R: A Language and Environment for Statistical Computing*. R Foundation for Statistical Computing. https://www.R-project.org/
